# The Earth Hologenome Initiative: Data Release 1

**DOI:** 10.1093/gigascience/giaf102

**Published:** 2025-09-05

**Authors:** Nanna Gaun, Carlotta Pietroni, Garazi Martin-Bideguren, Jonas Lauritsen, Ostaizka Aizpurua, Joana M Fernandes, Eduardo Ferreira, Fabien Aubret, Tom Sarraude, Constant Perry, Lucas Wauters, Claudia Romeo, Martina Spada, Claudia Tranquillo, Alex O Sutton, Michael Griesser, Miyako H Warrington, Guillem Pérez i de Lanuza, Javier Abalos, Prem Aguilar, Ferran de la Cruz, Javier Juste, Pedro Alonso-Alonso, Jim Groombridge, Rebecca Louch, Kevin Ruhomaun, Sion Henshaw, Carlos Cabido, Ion Garin Barrio, Emina Šunje, Peter Hosner, Ivan Prates, Geoffrey M While, Roberto García-Roa, Tobias Uller, Nathalie Feiner, Elisa Bonaccorso, Pernille Klein-Ipsen, Rosalina Molberg Rotovnik, Antton Alberdi, Raphael Eisenhofer

**Affiliations:** Center for Evolutionary Hologenomics, Globe Institute, University of Copenhagen, 1350 Copenhagen, Denmark; Center for Evolutionary Hologenomics, Globe Institute, University of Copenhagen, 1350 Copenhagen, Denmark; Center for Evolutionary Hologenomics, Globe Institute, University of Copenhagen, 1350 Copenhagen, Denmark; Center for Evolutionary Hologenomics, Globe Institute, University of Copenhagen, 1350 Copenhagen, Denmark; Center for Evolutionary Hologenomics, Globe Institute, University of Copenhagen, 1350 Copenhagen, Denmark; CESAM & Department of Biology, University of Aveiro, 3810-193 Aveiro, Portugal; CESAM & Department of Biology, University of Aveiro, 3810-193 Aveiro, Portugal; Station d'Ecologie Théorique et Expérimentale, CNRS, 09200 Moulis, France; Station d'Ecologie Théorique et Expérimentale, CNRS, 09200 Moulis, France; Station d'Ecologie Théorique et Expérimentale, CNRS, 09200 Moulis, France; Università degli Studi dell'Insubria, 21100 Varese, Italy; Center for Evolutionary Hologenomics, Globe Institute, University of Copenhagen, 1350 Copenhagen, Denmark; Istituto Zooprofilattico Sperimentale della Lombardia e dell'Emilia Romagna, 25124 Brescia, Italy; Università degli Studi dell'Insubria, 21100 Varese, Italy; Università degli Studi dell'Insubria, 21100 Varese, Italy; School of Environmental and Natural Sciences, Bangor University, Bangor LL57 2UR, UK; Department of Biology, University of Konstanz, 78464 Konstanz, Germany; Centre for the Advanced Study of Collective Behaviour, University of Konstanz, 78464 Konstanz, Germany; Department of Collective Behaviour, Max Planck Institute of Animal Behaviour, 78464 Konstanz, Germany; Luondua Boreal Research Station, 93391 Arvidsjaur, Sweden; Luondua Boreal Research Station, 93391 Arvidsjaur, Sweden; School of Biological and Medical Sciences, Oxford Brookes University, Headington, Oxford OX3 0BP, UK; Ethology Lab, Cavanilles Institute of Biodiversity and Evolutionary Biology, University of Valencia, 46980 Paterna, Valencia, Spain; Ethology Lab, Cavanilles Institute of Biodiversity and Evolutionary Biology, University of Valencia, 46980 Paterna, Valencia, Spain; Department of Biology, Lund University, 223 62 Lund, Sweden; Research Centre in Biodiversity and Genetic Resources, InBIO, CIBIO, Universidade do Porto, 4485-684 Vairão, Portugal; BIOPOLIS Program in Genomics, Biodiversity and Land Planning, CIBIO, 4485-661 Vairão, Portugal; Departamento de Biologia, Faculdade de Ciências da Universidade do Porto, 4169-007 Porto, Portugal; Research Centre in Biodiversity and Genetic Resources, InBIO, CIBIO, Universidade do Porto, 4485-684 Vairão, Portugal; Estación Biológica de Doñana (CSIC), 41092 Sevilla, Spain; Epidemiology and Public Health, CIBERESP, 28029 Madrid, Spain; Department of Animal Ecology and Tropical Biology, University of Würzburg, 97074 Würzburg, Germany; Durrell Institute of Conservation and Ecology, School of Natural Sciences, University of Kent, Kent, Canterbury CT2 7NZ, UK; Durrell Institute of Conservation and Ecology, School of Natural Sciences, University of Kent, Kent, Canterbury CT2 7NZ, UK; National Parks and Conservation Service, Ministry of Agro-Industry and Food Security, Government of Mauritius, RGP3+2RX Port Luis, Mauritius; Mauritian Wildlife Foundation, 73418 Vacoas-Phoenix, Mauritius; Aranzadi Science Foundation, 20014 Donostia-San Sebastián, Spain; Aranzadi Science Foundation, 20014 Donostia-San Sebastián, Spain; Faculty of Science, University of Sarajevo, 71000 Sarajevo, Bosnia and Herzegovina; Natural History Museum of Denmark, University of Copenhagen, 1350 Copenhagen, Denmark; Center for Global Mountain Biodiversity, University of Copenhagen, 2100 Copenhagen, Denmark; Center for Macroecology, Evolution, and Climate, University of Copenhagen, 2100 Copenhagen, Denmark; Department of Biology, Lund University, 223 62 Lund, Sweden; School of Natural Sciences, University of Tasmania, Dynnyrne TAS 7005, Australia; Department of Biology, Lund University, 223 62 Lund, Sweden; Department of Biology, Lund University, 223 62 Lund, Sweden; Department of Biology, Lund University, 223 62 Lund, Sweden; Max Planck Institute for Evolutionary Biology, 24306 Plön, Germany; Instituto Biósfera, Colegio de Ciencias Biológicas y Ambientales, Universidad San Francisco de Quito, 170901 Quito, Ecuador; Parasitology and Pathobiology, Department of Veterinary and Animal Sciences, University of Copenhagen, 1870 Copenhagen, Denmark; Parasitology and Pathobiology, Department of Veterinary and Animal Sciences, University of Copenhagen, 1870 Copenhagen, Denmark; Center for Evolutionary Hologenomics, Globe Institute, University of Copenhagen, 1350 Copenhagen, Denmark; Center for Evolutionary Hologenomics, Globe Institute, University of Copenhagen, 1350 Copenhagen, Denmark

**Keywords:** Bacteria, Genome, Genome-resolved metagenomics, MAG, Metagenome-assembled genome, Metagenomics, Microbiome, Microbiota

## Abstract

**Background:**

The Earth Hologenome Initiative (EHI) is a global endeavor dedicated to revisit fundamental ecological and evolutionary questions from the systemic host–microbiota perspective, through the standardized generation and analysis of joint animal genomic and associated microbial metagenomic data.

**Results:**

The first data release of the EHI contains 968 shotgun DNA sequencing read files containing 5.2 TB of raw genomic and metagenomic data derived from 21 vertebrate species sampled across 12 countries, as well as 17,666 metagenome-assembled genomes reconstructed from these data.

**Conclusions:**

The dataset can be used to address fundamental questions about host–microbiota interactions and will be available to the research community under the EHI data usage conditions.

## Background

The Earth Hologenome Initiative (EHI) [1] stands as a global scientific undertaking dedicated to revisit fundamental ecological and evolutionary questions from the systemic host–microbiota perspective [2, 3]. This goal is pursued through hologenomics, namely, the joint generation and analysis of host genomic and associated microbial metagenomic data [4]. The EHI unfolds through a 2-level approach with the participation of worldwide researchers representing >80 countries. At the initial level, the small- to medium-scale projects are executed, aiming to address taxon- or environment-specific scientific inquiries. While the sampling designs of each project are tailored to particular scientific questions, all projects follow standardized sample collection, metadata acquisition, and data generation procedures [5]. The second level leverages the inherent comparability of previously generated data to explore broad ecological and evolutionary questions requiring extensive taxonomic and geographical representation and larger amounts of data.

The EHI methodologies fully rely on DNA shotgun sequencing, enabling genome-wide analyses of animal hosts [6] and genome-resolved metagenomic analysis of their associated microbial communities [7]. Due to the primary interest in intestinal microbial communities, both data types are primarily sourced from fecal samples, which serve both as a proxy for lower intestinal microbial communities [8, 9] and as a useful data source for population genomic analyses [10]. Alternative sample types, such as blood and tissue samples, are also used when the amount of host DNA in feces is insufficient for host genome analyses. Occasionally, other sample types, such as skin or oral swabs, are also collected in the context of specific projects. Samples are usually obtained from live animals captured in the wild to ensure the collection of unaltered specimens along with relevant metadata about the host. The animals are released immediately after sampling.

This EHI data release includes raw DNA sequencing read files and metagenome-assembled genomes derived from these data [11]. All sequencing data are associated with a rich set of standardized metadata encompassing host phenotype, fieldwork, and laboratory information, which are required for the interpretation of the results.

## Data Description

### Context

**Figure 1:**
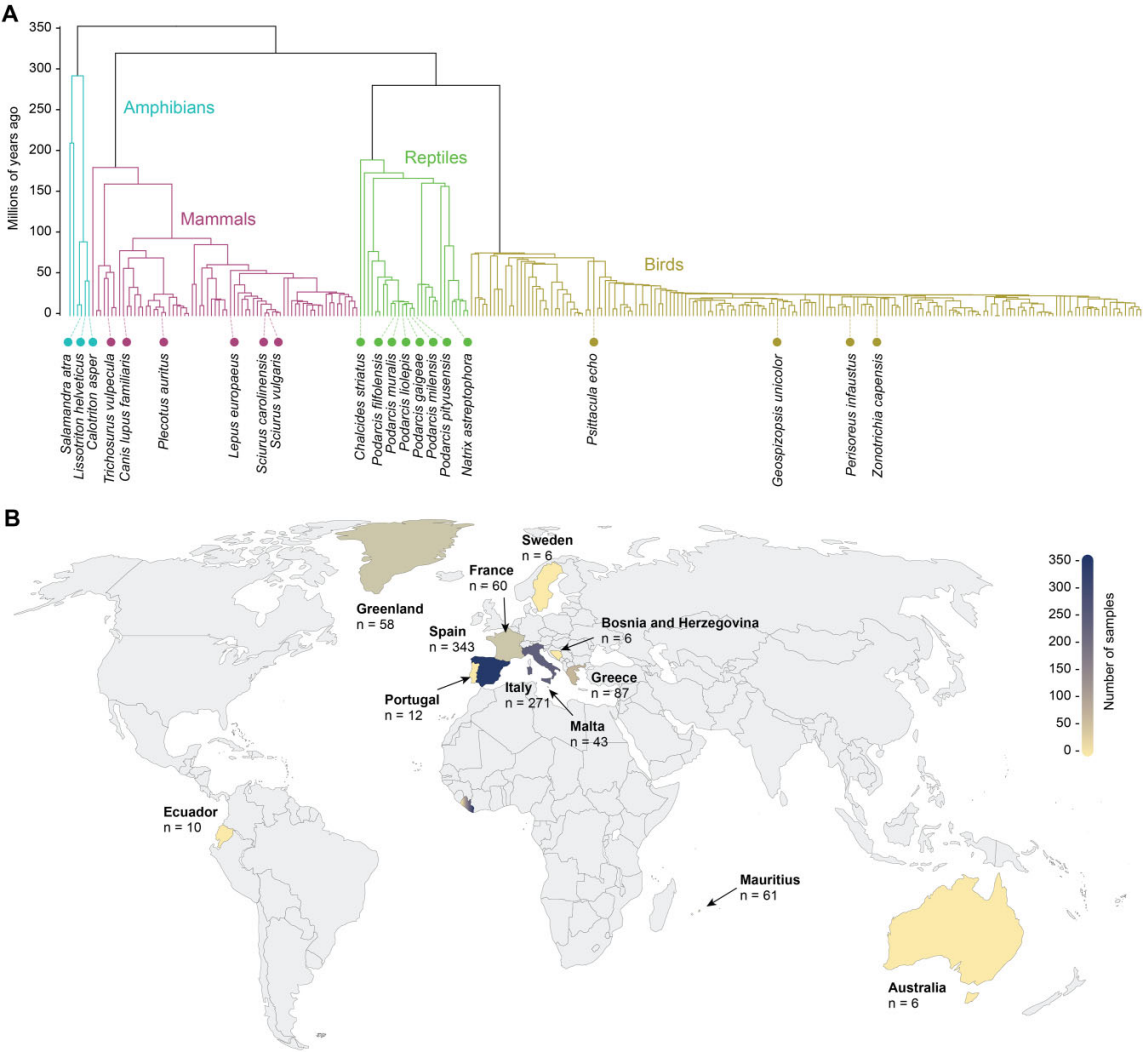
Phylogenetic placement and geographic origin of the samples. (A) Phylogenetic tree of all vertebrate species represented in the EHI collection in 2025 Q1, with the phylogenetic position of the species included in this data release highlighted. (B) World map indicating the number of samples sourced from each of the represented countries.

This first EHI data release contains raw sequencing data derived from 21 vertebrate species (Table 1). A total number of 902 samples were collected from animals across 317 sampling events that took place in 12 countries between January 2021 and December 2023 (Fig. 1). The sampling locations spanned 20 biomes, with most samples derived from temperate woodlands, followed by tropical forests, temperate shrublands, lakes or ponds, and polar tundra. All sampled specimens except the Greenland sled dogs (*Canis lupus familiaris*) were wild animals.

**Table 1:** Summary statistics of the animal species represented in the first EHI data release. Detailed metadata tables are available as part of the supporting files.

Species	Taxonomy	Sampling events	Individuals	Samples	Data (GB)	Microbial genomes	Percentage new
*Calotriton asper*	Urodela, Amphibia	5	31	37	230.4	745	95.0
*Canis lupus familiaris*	Carnivora, Mammalia	14	58	58	333.7	1,252	39.3
*Chalcides striatus*	Squamata, Reptilia	2	2	2	39.5	0	−
*Geospizopsis unicolor*	Passeriformes, Aves	1	2	2	18.3	0	−
*Lepus europaeus*	Lagomorpha, Mammalia	15	25	50	252.6	711	85.4
*Lissotriton helveticus*	Urodela, Amphibia	16	88	97	444.7	1,590	95.9
*Natrix astreptophora*	Squamata, Reptilia	2	2	2	32.8	0	−
*Perisoreus infaustus*	Passeriformes, Aves	2	2	2	32.5	0	−
*Plecotus auritus*	Chiroptera, Mammalia	1	2	2	42.1	0	−
*Podarcis filfolensis*	Squamata, Reptilia	9	43	43	174.7	693	91.9
*Podarcis gaigeae*	Squamata, Reptilia	17	61	61	303.5	1,280	97.3
*Podarcis liolepis*	Squamata, Reptilia	2	13	13	67.0	232	92.2
*Podarcis milensis*	Squamata, Reptilia	8	26	26	149.7	590	96.6
*Podarcis muralis*	Squamata, Reptilia	35	154	165	998.5	2,670	97.5
*Podarcis pityusensis*	Squamata, Reptilia	12	43	43	220.8	1,046	93.1
*Psittacula echo*	Psittaciformes, Aves	49	48	50	591.2	123	53.6
*Salamandra atra*	Urodela, Amphibia	1	2	2	23.8	0	−
*Sciurus carolinensis*	Rodentia, Mammalia	47	65	120	533.1	1,686	95.8
*Sciurus vulgaris*	Rodentia, Mammalia	76	74	123	660.5	1,033	72.3
*Trichosurus vulpecula*	Diprotodontia, Mammalia	2	2	2	20.9	61	88.5
*Zonotrichia capensis*	Passeriformes, Aves	1	2	2	28.0	0	−

Six different types of samples were processed: anal/cloacal swabs (*n* = 22), colon contents (*n* = 26), feces (*n* = 891), oral swabs (*n* = 13), skin swabs (*n* = 6), and skin tissue samples (*n* = 5). For a comparison of the quality of data generated from fecal and anal/cloacal swabs, see Pietroni et al. [5]. From these samples, 963 libraries were sequenced to yield 5,198 GB of data, with an average of 5.39 ± 3.84 GB per sample, representing 33% of the total data generated within the EHI until March 2025. The released data include 6.88% ± 7.14% of low-quality DNA, 16.57% ± 27.52% of DNA mapped to host genomes, and 76.54% ± 28.74% of metagenomic DNA.

**Figure 2:**
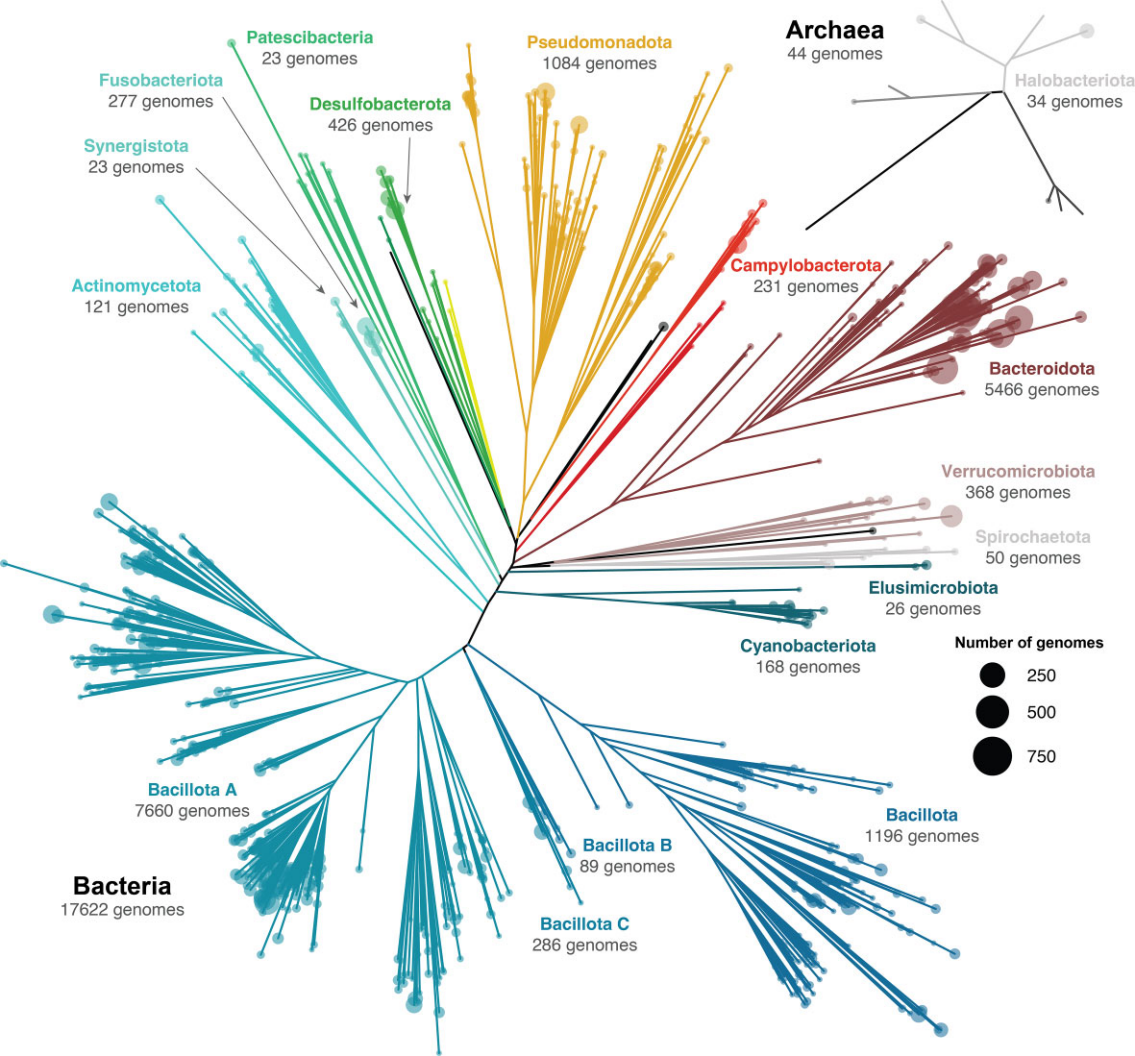
Phylogenetic trees of the EHI-reconstructed bacterial and archaeal genomes. Each tip represents a genus, and the tip size indicates the number of released genomes within the genus.

**Figure 3:**
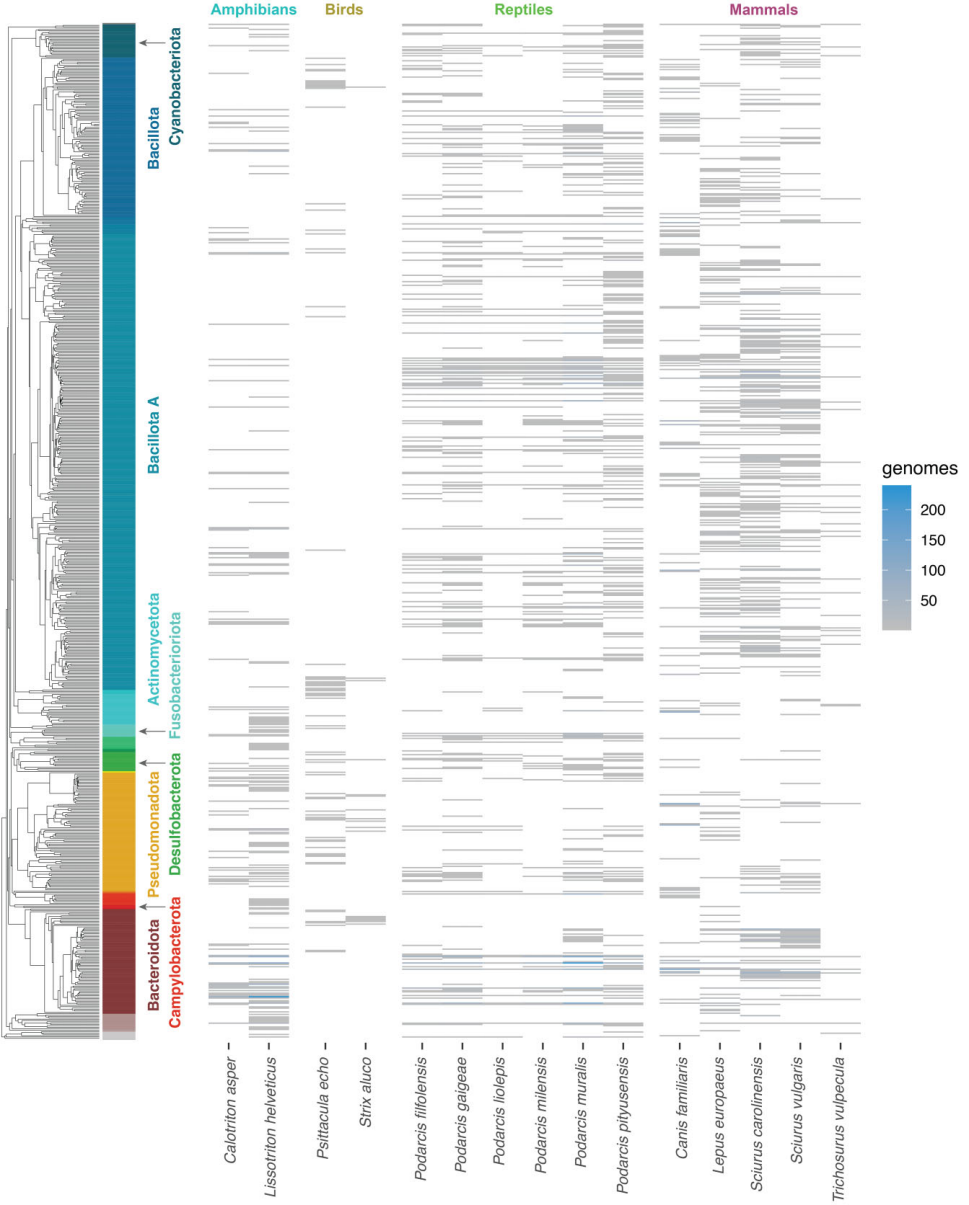
Host breadth of the reconstructed bacterial taxa. Only genomes reconstructed from individual assemblies are displayed in this figure. *Chalcides striatus, Geospizopsis unicolor, Natrix astreptophora, Plecotus auritus, Salamandra atra*, and *Zonotrichia capensis* did not yield any metagenome-assembled genomes from individual assemblies. Note that only the most abundant bacterial phylum names are displayed for the sake of visualization. Exact data can be found in the supplementary materials.

The current data release also includes 17,666 metagenome-assembled genomes (MAGs) derived from the binning of individual metagenomic assemblies conducted on the released sequencing data (Fig. 2). These MAGs derive from 15 different vertebrate species (Fig. 3) and have an average completeness value of 83.5% ± 15.3% and contamination value of 1.84% ± 2.07%. The catalog spans 33 phyla, with Bacillota A (7,660 MAGs) and Bacteroidota (5,466 MAGs) encompassing 73.9% of the reconstructed genomes. A total of 15,539 MAGs displayed an average nucleotide identity (ANI) below 95% with respect to any genome available at the R214 GTDB database [12], indicating an average novel species discovery rate of 87.9% [13]. All amphibian and reptile species displayed novel species discovery rates above 90%, with a maximum rate of 97.5%, as observed in the common wall lizard *Podarcis muralis* (Table 1).

### Methods

Data were generated using the standardized field, laboratory, and bioinformatic procedures implemented in the EHI, which are explained below.

#### Sample collection

Sample collection was conducted by the field scientists included in the author list, as specified in the Author Contributions section. Every field researcher received identical sampling guidelines and a standardized EHI sampling kit equipped with barcoded sample collection tubes containing 1 mL DNA/RNA Shield buffer (Zymo Research). In accordance with the manufacturer’s guidelines, a 1:10 sample-to-buffer ratio was employed, equating in the case of feces to approximately 100 mg of material. Adhering to EHI sample collection guidelines, samples were systematically accompanied by standardized metadata as outlined by Leonard et al. [1]. Most individual animals contributed at least 2 samples: fecal samples or anal/cloacal swabs were collected to generate gut microbial metagenomic data, while blood or tissue samples were collected to generate host genomic data when the host DNA in feces was insufficient for genome analysis. The samples were frozen within 2 weeks from collection, and details regarding sample preservation procedures prior to freezing were documented in the EHI database.

#### Laboratory processing

Laboratory sample processing was conducted at the Globe Institute’s (University of Copenhagen) molecular laboratory in Copenhagen, Denmark, following the established EHI laboratory protocols [14]. In summary, samples underwent bead-beating before DNA isolation employing silica magnetic beads (G-Biosciences) with solid-phase reversible immobilization. The concentration of DNA extracts was quantified through a Qubit 3 Fluorometer (Thermo Fisher Scientific) using dsDNA HS (High Sensitivity) Assay Kits (Thermo Fisher Scientific). Subsequently, DNA was fragmented into approximately 450-bp-long fragments using a Covaris LE220 platform (Covaris). Library preparation followed the ligation-based BEST protocol [15], utilizing a standard input of 200 ng DNA in 24 µL or the closest amount feasible based on the sample DNA concentration. We used 1.5 µL of 20 µM adapters for a 50- to 200-ng DNA input, 1.5 µL of 10 µM for 10–50 ng, 1.5 µL of 5 µM for <10 ng, and 1.5 µL of 2 µM for samples below the quantification range. Libraries underwent quantitative PCR screening to determine the optimal number of library indexing PCR cycles [16], followed by PCR amplification using unique dual index primers with an adjusted number of cycles. The resulting libraries underwent automated capillary electrophoresis using Fragment Analyzer (Agilent) for assessment of fragment-length distribution, adapter dimers, and adapter-to-library molar ratios. Finally, samples were pooled into 21 sequencing batches, and sequencing was performed across multiple lanes of NovaSeq6000 (RRID:SCR_016387) and NovaSeq X (RRID:SCR_024569) platforms (Illumina), generating an average of 5 GB (approximately 16.6 million reads) of 150-bp paired-end sequencing data per sample.

#### Bioinformatics

The raw sequencing data underwent processing through the automated EHI bioinformatic pipeline [17], which is briefly explained below. The raw, intermediate, and final data were archived in the Electronic Research Data Archive at the University of Copenhagen [18]. Meanwhile, sample locations and pertinent metadata were stored in the EHI Database, built upon the Airtable software. Computation tasks were executed on the local cluster of the Globe Institute (Mjolnir), using custom bioinformatic pipelines based on Snakemake (RRID:SCR_003475) [19] and executed through slurm [20].

In the preprocessing step, fastp [21] was employed for quality filtering, followed by alignment against the reference host genome using Bowtie2 (RRID:SCR_016368) [22]. Mapped reads were retained for genomic analyses, while unmapped reads were isolated using SAMTOOLS (RRID:SCR_002105) [23] for subsequent metagenomic analyses. The unmapped fraction underwent complexity analysis using Nonpareil 3 [24] and microbial fraction estimation using SingleM [25, 26]. Subsequently, metagenomic assemblies were conducted for each individual sample using MEGAHIT v1.2.9 [27], followed by binning using CONCOCT [28], MaxBin2 [29], and MetaBAT2 [30]. Assembly statistics were generated using QUAST v5.2.0 [31]. The bins were subsequently refined using MetaWRAP’s refinement module [32] with CheckM [33]. Taxonomic annotation utilized GTDB-tk v2.3.0 [12] against the R214 GTDB database [34], and the phylogenetic tree of MAGs was constructed by pruning the reference genomes using drop.tip function of the ape R package [35].

#### Data archiving

Raw sequencing data (FASTQ format) were archived at the European Nucleotide Archive (ENA), while draft bacterial genomes (FASTA format) were compiled in a tarball file and archived in Zenodo. We also offer users the option to obtain download links to specific MAGs directly from the EHI database [36]. Metadata specific to this data release, as well as the code used for visualization and summary statistics, are stored in GitHub, with a release frozen in Zenodo. Relevant URLs, DOIs, and accession numbers are mentioned in the Data Availability section.

### Data validation and quality control

We implemented numerous measures in the field, laboratory, and bioinformatic procedures to ensure that the generated data were representative of the collected biological samples and comparable across samples obtained by different field researchers across the world [37], as detailed below.

#### Field quality control

The quality control measures implemented in the field included the usage of standardized sampling kits and guidelines to ensure all samples were collected following identical procedures. All field researchers were informed about the sensitivity of shotgun sequencing procedures regarding environmental contamination and cross-contamination, thus requiring them to employ clean items for storing and manipulating the animals and the samples, using protective synthetic gloves and continuously sterilizing tools. Samples were frozen at or below −18°C, ideally within a day and at maximum within the first 2 weeks after sample collection. Time until freezing was recorded as one of the technical metadata variables.

#### Laboratory quality control

All sampling tubes were prelabeled with identical human-readable (5-digit code with 3 letters and 2 numbers; e.g., ABC99) and machine-readable (QR code) barcodes. Upon arrival at the Globe Institute, samples and metadata sheets were cross-checked and inconsistencies addressed before indexing the samples in the EHI database. This manual quality control also included logging deviations from standard procedures (e.g., overstuffing tubes with sample material) and technical issues such as leaking of sample tubes, which resulted in the disposal of unsuitable samples. All DNA extraction batches included blanks to monitor contamination and were organized according to expected DNA yield to minimize cross‐contamination. Due to the variability of sample sources and types, concentrations of all DNA extracts were measured using a Qubit 3 Fluorometer, both to adjust the volumes for library preparation and to account for DNA template amount in statistical analyses. Sequencing adapter molarities were adjusted to the amount of input DNA to minimize the formation of adapter dimers and other artifacts, and all libraries were screened through quantitative PCR (Mx3005p; Agilent) to assess library preparation success and tailor the number of required indexing PCR cycles to each library. All indexed libraries were analyzed through capillary electrophoresis for high-quality measurement of library molarities, to ensure the required amount of sequencing data was generated.

#### Bioinformatic quality control

We employed multiple criteria to assess the quality and representativeness of the generated data. Following standard quality filtering, we removed reads with average phred-scores below q30 (1 sequencing error expected every 1,000 bases) and trimmed reads with low-quality endings and adapter remnants. To further assess library preparation success, we estimated duplication rates using the reads mapped to the host reference genome. Unmapped reads were further screened for complexity using Nonpareil 3, and the microbial read fraction was estimated using SingleM. Through all these measurements, we estimated expected levels of diversity and complexity, which we then used to assess the representativeness of the generated MAGs. Following field standards [38], only bins exceeding 50% completeness and maintaining contamination levels below 10% were considered MAGs to be included in downstream analyses.

### Ethics

The EHI is governed by open science principles, adhering to CARE and FAIR data governance frameworks [12, 13], as well as complying with all international, national, and regional regulations stemming from the United Nations’ Convention on Biological Diversity [39]. In line with these commitments, the rights and interests of Indigenous peoples are fully considered by actively involving local scientists in research projects. These scientists co-own the samples collected within the EHI framework, as well as the data derived from them. All sample collection, exportation, and data generation strictly adhere to local and international legislation on access and benefit-sharing (ABS) of genetic resources, as outlined in the Nagoya Protocol and implemented through national ABS laws. Accordingly, all sampling, material transfer, and ABS permits are filed in the EHI database. Finally, this data release serves as a testament to our commitment to making the data findable, accessible, interoperable, and reusable (FAIR), ensuring its maximum research and societal impact.

### Reuse potential

The Earth Hologenome Initiative was established to promote high-quality, open hologenomic research on wild animals and their associated microorganisms. This data release, like those to follow, reflects our commitment to fostering collective efforts to understand and conserve biodiversity on our planet. Following the norms set in the Bermuda Principles, Fort Lauderdale agreement, and Toronto International Data Release Workshop [40], the authors kindly request users to respect the rights of the many researchers who invested significant effort in collecting samples and generating data for primary research. For 1 year following this article’s publication, anyone wishing to use these data to investigate animal or microbial ecological and evolutionary questions should first contact the corresponding author. Following this communication, the EHI Management will facilitate discussions between interested users and the original researchers to ensure efforts are coordinated with the people that are already working with these data.

## Availability of Source Code and Requirements

Project name: Earth Hologenome Initiative Data Release 1

Project homepage: https://github.com/earthhologenome/EHI_data_release_1

Operating system(s): Platform independent

Programming language: R

License: CC0

## Abbreviations

ABS: access and benefit-sharing; ANI: average nucleotide identity; EHI: Earth Hologenome Initiative; ENA: European Nucleotide Archive; FAIR: findable, accessible, interoperable, and reusable; GB: gigabases; MAG: metagenome-assembled genome.

## Supplementary Material

giaf102_Authors_Response_To_Reviewer_Comments_Original_Submission

giaf102_Authors_Response_To_Reviewer_Comments_Revision_1

giaf102_GIGA-D-25-00196_original_submission

giaf102_GIGA-D-25-00196_Revision_1

giaf102_GIGA-D-25-00196_Revision_2

giaf102_Reviewer_1_Report_Original_Submission Chuen Zhang Lee -- 6/6/2025

giaf102_Reviewer_2_Report_Original_Submission Yendi E Navarro Noya -- 7/30/2025

## Data Availability

Raw sequencing data belonging to the first EHI data release are available at the European Nucleotide Archive, under Bioproject accession number PRJEB76898, which is nested within the Earth Hologenome Initiative’s umbrella Bioproject PRJEB51837. A tarball containing fasta files of all MAGs was deposited in Zenodo [41]. Details of the specific sample and data accession numbers, their associated metadata, and the code used for visualization and summary statistics can be found in GitHub [42], with a snapshot in Zenodo [43]. In addition, the GitHub repository is also archived in Software Heritage [44]. The overview of all EHI data is available at the EHI database [36].
