# Supplementary material for: The Earth Hologenome Initiative: Data Release 1
Source: Gigascience. 2025 Sep 5;14:giaf102. doi: 10.1093/gigascience/giaf102 (PMC12412122; doi:10.1093/gigascience/giaf102)

GigaScience

The Earth Hologenome Initiative: Data Release 1

--Manuscript Draft--

Manuscript Number:	GIGA-D-25-00196R2
Full Title:	The Earth Hologenome Initiative: Data Release 1
Article Type:	Data Note
Funding Information:	Danmarks Grundforskningsfond (DNRF143) — Dr Antton Alberdi<br>Carlsbergfondet (CF20-0460) — Dr Antton Alberdi<br>HORIZON EUROPE Framework Programme (101066225) — Dr Claudia Romeo<br>Agência Regional para o Desenvolvimento da Investigação, Tecnologia e Inovação (PD/BD/150645/2020) — Ms Joana Fernandes<br>Villum Fonden (25925) — Dr Peter Hosner
Abstract:	Background<br>The Earth Hologenome Initiative (EHI) is a global endeavour dedicated to revisit fundamental ecological and evolutionary questions from the systemic host-microbiota perspective, through the standardised generation and analysis of joint animal genomic and associated microbial metagenomic data.<br>Results<br>The first data release of the EHI contains 968 shotgun DNA sequencing read files containing 5.2 TB of raw genomic and metagenomic data derived from 21 vertebrate species sampled across 12 countries, as well as 17,666 metagenome-assembled genomes (MAGs) reconstructed from these data.<br>Conclusions<br>The dataset can be used to address fundamental questions about host-microbiota interactions, and become available to the research community under the EHI data usage conditions.
Corresponding Author:	Antton Alberdi<br>University of Copenhagen: Kobenhavns Universitet<br>Copenhagen, DENMARK
Corresponding Author Secondary Information:	
Corresponding Author's Institution:	University of Copenhagen: Kobenhavns Universitet
Corresponding Author's Secondary Institution:	
First Author:	Nanna Gaun
First Author Secondary Information:	
Order of Authors:	Nanna Gaun
	Carlotta Pietroni
	Garazi Martin-Bideguren
	Jonas Grev Lauritsen
	Ostaizka Aizpurua
	Joana Fernandes
	Eduardo Ferreira
	Fabien Aubret

Powered by Editorial Manager® and ProduXion Manager® from Aries Systems Corporation

	Tom Sarraude
	Constant Perry
	Lucas Wauters
	Claudia Romeo
	Martina Spada
	Claudia Tranquillo
	Alex O Sutton
	Michael Griesser
	Miyako H Warrington
	Guillem Pérez i de Lanuza
	Javier Avalos
	Prem Aguilar
	Ferran de la Cruz
	Javier Juste
	Pedro Alonso-Alonso
	Jim Groombridge
	Rebecca Louch
	Kevin Ruhomaun
	Sion Henshaw
	Carlos Cabido
	Ion Garin Barrio
	Emina Šunje
	Peter Hosner
	Ivan Prates
	Geoffrey M While
	Roberto García-Roa
	Tobias Uller
	Nathalie Feiner
	Elisa Bonaccorso
	Pernille Klein-Ipsen
	Rosalina Rotovnik
	Antton Alberdi
	Raphael Eisenhofer
**Order of Authors Secondary Information:**	
**Response to Reviewers:**	The updated manuscript file contains all the requested edits.
**Additional Information:**	
**Question**	**Response**
Are you submitting this manuscript to a special series or article collection?	No

Powered by Editorial Manager® and ProduXion Manager® from Aries Systems Corporation

**Experimental design and statistics**<br><br>Full details of the experimental design and statistical methods used should be given in the Methods section, as detailed in our Minimum Standards Reporting Checklist. Information essential to interpreting the data presented should be made available in the figure legends.<br><br>Have you included all the information requested in your manuscript?	No
If not, please give reasons for any omissions below.<br><br>as follow-up to "**Experimental design and statistics**<br><br>Full details of the experimental design and statistical methods used should be given in the Methods section, as detailed in our Minimum Standards Reporting Checklist. Information essential to interpreting the data presented should be made available in the figure legends.<br><br>Have you included all the information requested in your manuscript?<br><br>"	The manuscript is a data note without statistical tests.
**Resources**<br><br>A description of all resources used, including antibodies, cell lines, animals and software tools, with enough information to allow them to be uniquely identified, should be included in the Methods section. Authors are strongly encouraged to cite Research Resource Identifiers (RRIDs) for antibodies, model organisms and tools, where possible.<br><br>Have you included the information	Yes

requested as detailed in our Minimum Standards Reporting Checklist?	
**Availability of data and materials**<br><br>All datasets and code on which the conclusions of the paper rely must be either included in your submission or deposited in publicly available repositories (where available and ethically appropriate), referencing such data using a unique identifier in the references and in the “Availability of Data and Materials” section of your manuscript.<br><br>Have you have met the above requirement as detailed in our Minimum Standards Reporting Checklist?	Yes
GigaScience has policies and guidelines in place for the use of generative AI-writing tools such as ChatGPT. If you have used such writing tools to assist with writing the manuscript this must be declared and cited in the text. Authors should not list AI-writing tools and other AI-assisted technologies as an author or co-author and should acknowledge that they are fully responsible for text generated or refined by AI-writing tools.<p><br><br>A summary of use (particularly in the introduction or among methods) needs to be included at the end of the paper, and the outputs should also be included as a supplementary file hosted in GigaDB or other open repositories. Please read our guidelines for more information. <p><br><br>By submitting to GigaScience, you are aware of the journal's AI-writing tools policy, and if you have declared use of such tools below, you have acknowledged this where appropriate in your manuscript and have made a summary of use and outputs available. <p><br><b>AI-assisted writing tools have been used in the preparation of this manuscript?	No

Manuscript

Click here to access/download;Manuscript;EHI_data_release_1_v3 (1).docx

The Earth Hologenome Initiative: Data Release 1

Nanna Gaun[1], Carlotta Pietroni[1], Garazi Martin-Bideguren[1], Jonas Lauritsen[1], Ostaizka Aizpurua[1], Joana M Fernandes[2], Eduardo Ferreira[2], Fabien Aubret[3], Tom Sarraude[3], Constant Perry[3], Lucas Wauters[4], Claudia Romeo[1,5], Martina Spada[4], Claudia Tranquillo[4], Alex O Sutton[6], Michael Griesser[7,8,9,10], Miyako H Warrington[10,11], Guillem Pérez i de Lanuza[12], Javier Abalos[12,13], Prem Aguilar[14], Ferran de la Cruz[14], Javier Juste[15,16], Pedro Alonso-Alonso[17], Jim Groombridge[18], Rebecca Louch[18], Kevin Ruhomaun[19], Sion Henshaw[20], Carlos Cabido[21], Ion Garin Barrio[21], Emina Šunje[22], Peter Hosner[23,24,25], Ivan Prates[13], Geoffrey M While[26], Roberto García-Roa[13], Tobias Uller[13], Nathalie Feiner[13,27], Elisa Bonaccorso[28], Pernille Klein-Ipsen[29], Rosalina Rotovnik[29], Antton Alberdi[1]*, and Raphael Eisenhofer[1]

[1] Center for Evolutionary Hologenomics, Globe Institute, University of Copenhagen, Denmark.
[2] CESAM & Department of Biology, University of Aveiro, Aveiro, Portugal.
[3] Station d'Ecologie Théorique et Expérimentale, CNRS.
[4] Università degli Studi dell'Insubria, Varese, Italy.
[5] Istituto Zooprofilattico Sperimentale della Lombardia e dell’Emilia Romagna, Brescia, Italy.
[6] School of Environmental and Natural Sciences, Bangor University.
[7] Department of Biology, University of Konstanz, Konstanz, Germany.
[8] Centre for the Advanced Study of Collective Behaviour, University of Konstanz, Konstanz, Germany.
[9] Department of Collective Behaviour, Max Planck Institute of Animal Behaviour, Konstanz, Germany.
[10] Luondu Boreal Research Station, Arvidsjaur, Sweden.
[11] School of Biological and Medical Sciences, Oxford Brookes University, Headington, OX3 0BP, UK.
[12] Ethology Lab, Cavanilles Institute of Biodiversity and Evolutionary Biology, University of Valencia, Spain.
[13] Department of Biology, Lund University, Sweden.
[14] Research Centre in Biodiversity and Genetic Resources, InBIO, CIBIO, Universidade do Porto, Porto, Portugal.
[15] Estación Biológica de Doñana (CSIC), Sevilla, Spain.
[16] Epidemiology and Public Health, CIBERESP, Madrid, Spain.
[17] Department of Animal Ecology and Tropical Biology. University of Würzburg, Würzburg, Germany.
[18] Durrell Institute of Conservation and Ecology, School of Natural Sciences, University of Kent, UK.
[19] National Parks and Conservation Service, Ministry of Agro-Industry and Food Security, Government of Mauritius.
[20] Mauritian Wildlife Foundation, Vacoas, Mauritius.
[21] Aranzadi Science Foundation, Donostia-San Sebastián.
[22] University of Sarajevo, Sarajevo, Serbia.
[23] Natural History Museum of Denmark, University of Copenhagen, Denmark.

[24] Center for Global Mountain Biodiversity, University of Copenhagen, Denmark.
[25] Center for Macroecology, Evolution, and Climate, University of Copenhagen, Denmark.
[26] School of Natural Sciences, University of Tasmania, Australia.
[27] Max Planck Institute for Evolutionary Biology, Plön, Germany.
[28] Instituto Biósfera, Colegio de Ciencias Biológicas y Ambientales, Universidad San Francisco de Quito, Quito, Ecuador
[29] Parasitology and Pathobiology, Department of Veterinary and Animal Sciences, University of Copenhagen, Denmark.

*Correspondence: antton.alberdi@sund.ku.dk

Abstract

Background

The Earth Hologenome Initiative (EHI) is a global endeavour dedicated to revisit fundamental ecological and evolutionary questions from the systemic host-microbiota perspective, through the standardised generation and analysis of joint animal genomic and associated microbial metagenomic data.

Results

The first data release of the EHI contains 968 shotgun DNA sequencing read files containing 5.2 TB of raw genomic and metagenomic data derived from 21 vertebrate species sampled across 12 countries, as well as 17,666 metagenome-assembled genomes (MAGs) reconstructed from these data.

Conclusions

The dataset can be used to address fundamental questions about host-microbiota interactions, and become available to the research community under the EHI data usage conditions.

Background

The Earth Hologenome Initiative (EHI) [1] stands as a global scientific undertaking dedicated to revisit fundamental ecological and evolutionary questions from the systemic host-microbiota perspective [2,3]. This goal is pursued through hologenomics, namely the joint generation and analysis of host genomic and associated microbial metagenomic data [4]. The EHI unfolds through a two-level approach with the participation of worldwide researchers representing >80 countries. At the initial level, the small- to medium-scale projects are executed, aiming to address taxon- or environment-specific scientific inquiries. While the sampling designs of each project are tailored to particular scientific questions, all projects follow standardised sample collection, metadata acquisition, and data generation procedures [5]. The second level leverages the inherent comparability of previously generated data to explore broad ecological and evolutionary questions requiring extensive taxonomic and geographical representation and larger amounts of data.

The EHI methodologies fully rely on DNA shotgun sequencing, enabling genome-wide analyses of animal hosts [6] and genome-resolved metagenomic analysis of their associated microbial communities [7]. Due to the primary interest in intestinal microbial communities, both data types are primarily sourced from faecal samples, which serve both as a proxy for lower intestinal microbial communities [8,9], as well as a useful data source for population genomic analyses [10]. Alternative sample types, such as blood and tissue samples, are also used when the amount of host DNA in faeces is insufficient for host genome analyses. Occasionally, other sample types such as skin or oral swabs are also collected in the context of specific projects. Samples are usually obtained from live animals captured in the wild to ensure the collection of unaltered specimens along with relevant metadata about the host. The animals are released immediately after sampling.

This EHI data release includes raw DNA sequencing read files, and metagenome-assembled genomes derived from these data [11]. All sequencing data are associated with a rich set of standardised metadata encompassing host phenotype, fieldwork and laboratory information, which are required for the interpretation of the results.

Data description

Context

This first EHI data release contains raw sequencing data derived from 21 vertebrate species (Table 1). A total number of 902 samples were collected from animals across 317 sampling events that took place in 12 countries between January 2021 and December 2023 (Figure 1). The sampling locations spanned 20 biomes, with most samples derived from temperate woodlands, followed by tropical forests, temperate shrublands, lakes or ponds, and polar tundra. All sampled specimens except the Greenland sled dogs (*Canis lupus familiaris*) were wild animals.

Six different types of samples were processed: anal/cloacal swabs (n=22), colon contents (n=26), faeces (n=891), oral swabs (n=13), skin swabs (n=6) and skin tissue samples (n=5). For a comparison of the quality of data generated from faecal and anal/cloacal swabs see Pietroni et al. (2025). From these samples, 963 libraries were sequenced to yield 5,198 gigabases (GB) of data, with an average of 5.39±3.84 GB per sample, representing 33% of the total data generated within the EHI until March 2025. The released data include 6.88±7.14% of low-quality DNA, 16.57±27.52% of DNA mapped to host genomes, and 76.54±28.74% of metagenomic DNA.

The current data release also includes 17,666 metagenome-assembled genomes (MAGs) derived from the binning of individual metagenomic assemblies conducted on the released sequencing data (Figure 2). These MAGs derive from 15 different vertebrate species (Figure 3), have an average completeness value of 83.5±15.3% and contamination value of 1.84±2.07%. The catalogue spans 33 phyla, with Bacillota A (7660 MAGs), and Bacteroidota (5466 MAGs) encompassing 73.9% of the reconstructed genomes. A total of 15,539 MAGs displayed an average nucleotide identity (ANI) below 95% with respect to any genome available at the R214 GTDB database [12], indicating an average novel species discovery rate of 87.9% [13]. All amphibian and reptile species displayed novel species discovery rates above 90%, with a maximum rate of 97.5% as observed in the common wall lizard *Podarcis muralis* (Table 1).

Methods

Data were generated using the standardised field, laboratory, and bioinformatic procedures implemented in the EHI, which are explained below.

Sample collection

Sample collection was conducted by the field scientists included in the author list, as specified in the author contributions section. Every field researcher received identical sampling guidelines and a standardised EHI sampling kit equipped with barcoded sample collection tubes containing 1 ml of DNA/RNA Shield buffer (Zymo Research, USA). In accordance with the manufacturer's guidelines, a 1:10 sample-to-buffer ratio was employed, equating in the case of faeces to approximately 100 mg of material. Adhering to EHI sample collection guidelines, samples were systematically accompanied by standardised metadata as outlined by Leonard et al.~~ (2024)~~ [1]. Most individual animals contributed at least two samples: faecal samples or anal/cloacal swabs were collected to generate gut microbial metagenomic data, while blood or tissue samples were collected to generate host genomic data when the host DNA in faeces was insufficient for genome analysis. The samples were frozen within two weeks from collection, and details regarding sample preservation procedures prior to freezing were documented in the EHI database.

Laboratory processing

Laboratory sample processing was conducted at the Globe Institute's (University of Copenhagen) molecular laboratory in Copenhagen, Denmark, following the established EHI laboratory protocols [14]~~available at www.earthhologenome.org/laboratory~~. In summary, samples underwent bead-beating before DNA isolation employing silica magnetic beads (G-Biosciences, USA) with solid-phase reversible immobilisation. The concentration of DNA extracts was quantified through a Qubit™ 3 Fluorometer (Thermo Fisher Scientific, USA) using dsDNA HS (High Sensitivity) Assay Kits. Subsequently, DNA was fragmented into approximately 450 bp-long fragments using a Covaris LE220 platform (Covaris, USA). Library preparation followed the ligation-based BEST protocol [15], utilising a standard input of 200 ng of DNA in 24 µl or the closest amount feasible based on the sample DNA concentration. We used 1.5 µl of 20 µM adaptors for a 50-200 ng DNA input, 1.5 µl of 10 µM for 10-50 ng, 1.5 µl of 5 µM for <10 ng, and 1.5 µl of 2 µM for samples below the quantification range. Libraries underwent qPCR screening to determine the optimal number of library indexing PCR cycles [16], followed by PCR amplification using unique dual index primers with an adjusted number of cycles. The resulting libraries underwent automated capillary electrophoresis using Fragment Analyzer (Agilent, USA) for assessment of fragment-length distribution, adaptor dimers, and adaptor-to-library molar ratios. Finally, samples were pooled into 21 sequencing batches, and sequencing was performed across multiple lanes of NovaSeq6000 (RRID:SCR_016387) and NovaSeq X (RRID:SCR_024569) platforms (Illumina, USA), generating an average of 5 GB (approximately 16.6 million reads) of 150 bp paired-end sequencing data per sample.

Bioinformatics

The raw sequencing data underwent processing through the automated EHI bioinformatic pipeline [17], which is~~accessible at www.earthhologenome.org/bioinformatics, and~~ briefly explained below. The raw, intermediate, and final data were archived in the Electronic Research Data Archive ~~(ERDA; www.erda.dk) ~~at the University of Copenhagen [18]. Meanwhile, sample

locations, and pertinent metadata were stored in the EHI Database, built upon the Airtable software (Airtable, USA). Computation tasks were executed on the local cluster of the Globe Institute (Mjolnir), using custom bioinformatic pipelines based on S~~s~~nakemake (RRID:SCR_003475) [19] and executed through slurm [20].

In the preprocessing step, fastp [21] was employed for quality filtering, followed by alignment against the reference host genome using Bowtie2 (RRID:SCR_016368) [22]. Mapped reads were retained for genomic analyses, while unmapped reads were isolated using SAMTOOLS~~samtools~~ (RRID:SCR_002105) [23] for subsequent metagenomic analyses. The unmapped fraction underwent complexity analysis using Nonpareil 3 [24] and microbial fraction estimation using SingleM [25,26]. Subsequently, metagenomic assemblies were conducted for each individual sample using MEGAHIT v1.2.9 [27], followed by binning using CONCOCT [28], MaxBin2 [29], and MetaBAT2 [30]. Assembly statistics were generated using QUAST v5.2.0 [31]. The bins were subsequently refined using MetaWRAP's refinement module [32] with CheckM [33]. Taxonomic annotation utilised GTDB-tk v2.3.0 [12] against the R214 GTDB database [34], and the phylogenetic tree of MAGs was constructed by pruning the reference genomes using drop.tip function of the ape R package [35].

Data archiving

Raw sequencing data (FASTQ format) was archived at the European Nucleotide Archive (ENA), while draft bacterial genomes (FASTA format) were compiled in a tarball file and archived in Zenodo. We also offer users the option to obtain download links to specific MAGs directly from the EHI database [36]~~(https://www.earthhologenome.org/database)~~. Metadata specific to this data release, as well as the code used for visualisation and summary statistics are stored in Github, with a release frozen in Zenodo. Relevant URLs, DOIs, and accession numbers are mentioned in the Data Availability section.

Data validation and quality control

We implemented numerous measures in the field, laboratory, and bioinformatic procedures to ensure that the generated data were representative of the collected biological samples and comparable across samples obtained by different field researchers across the world [37], as detailed below.

Field quality-control

The quality-control measures implemented in the field included the usage of standardised sampling kits and guidelines to ensure all samples were collected following identical procedures. All field researchers were informed about the sensitivity of shotgun sequencing procedures regarding environmental contamination and cross-contamination, thus requiring them to employ clean items for storing and manipulating the animals and the samples, using protective synthetic gloves and continuously sterilising tools. Samples were frozen at or below -18ºC, ideally within a day and at maximum within the first two weeks after sample collection. Time until freezing was recorded as one of the technical metadata variables.

Laboratory quality-control

All sampling tubes were pre-labelled with identical human- (5-digit code with 3 letters and 2 numbers; e.g., ABC99) and machine-readable (QR code) barcodes. Upon arrival at the Globe Institute, samples and metadata sheets were cross-checked and inconsistencies addressed before indexing the samples in the EHI database. This manual quality-control also included logging deviations from standard procedures (e.g. overstuffing tubes with sample material), and technical issues such as leaking of sample tubes, which resulted in the disposal of unsuitable samples. All DNA extraction batches included blanks to monitor contamination and were organised according to expected DNA yield to minimise cross- contamination. Due to the variability of sample sources and types, concentrations of all DNA extracts were measured using a Qubit™ 3 Fluorometer, both to adjust the volumes for library preparation and to account for DNA template amount in statistical analyses. Sequencing adaptor molarities were adjusted to the amount of input DNA to minimise the formation of adaptor dimers and other artefacts, and all libraries were screened through qPCR (Mx3005p, Agilent, USA) to assess library preparation success and tailor the number of required indexing PCR cycles to each library. All indexed libraries were analysed through capillary electrophoresis for high-quality measurement of library molarities, to ensure the required amount of sequencing data was generated.

Bioinformatic quality-control

We employed multiple criteria to assess the quality and representativeness of the generated data. Following standard quality filtering, we removed reads with average phred-scores below q30 (one sequencing error expected every 1000 bases), and trimmed reads with low-quality endings and adaptor remnants. To further assess library preparation success, we estimated duplication rates using the reads mapped to the host reference genome. Unmapped reads were further screened for complexity using Nonpareil 3, and the microbial read fraction was estimated using SingleM. Through all these measurements we estimated expected levels of diversity and complexity, which we then used to assess the representativeness of the generated MAGs. Following field standards [38], only bins exceeding 50% completeness and maintaining contamination levels below 10% were considered as MAGs to be included in downstream analyses.

Ethics

The EHI is governed by open science principles, adhering to CARE and FAIR data governance frameworks [12,13], and complying with all international, national and regional regulations stemming from the United Nations' Convention on Biological Diversity (www.cbd.int). In line with these commitments, the rights and interests of Indigenous peoples are fully considered by actively involving local scientists in research projects. These scientists co-own the samples collected within the EHI framework, as well as the data derived from them. All sample collection, exportation, and data generation strictly adhere to local and international legislations on access and benefit-sharing (ABS) of genetic resources, as outlined in the Nagoya Protocol and implemented through national ABS laws. Accordingly, all sampling, material transfer, and ABS permits are filed in the EHI database. Finally, this data release serves as a testament to our

commitment to making the data findable, accessible, interoperable, and reusable (FAIR), ensuring its maximum research and societal impact.

Re-use potential

The Earth Hologenome Initiative was established to promote high-quality, open hologenomic research on wild animals and their associated microorganisms. This data release, like those to follow, reflects our commitment to fostering collective efforts to understand and conserve biodiversity on our planet. Following the norms set in the Bermuda Principles, Fort Lauderdale agreement and Toronto International Data Release Workshop [39], the authors kindly request users to respect the rights of the many researchers who invested significant effort in collecting samples and generating data for primary research. For one year following this manuscript's publication, anyone wishing to use these data to investigate animal or microbial ecological and evolutionary questions should first contact the corresponding author. Following this communication, the EHI Management will facilitate discussions between interested users and the original researchers to ensure efforts are coordinated with the people that are already working with these data.

Availability of source code and requirements

Project name: Earth Hologenome Initiative Data Release 1

Project home page: https://github.com/earthhologenome/EHI_data_release_1

Operating system(s): Platform independent

Programming language: R

License: CC0

Formatted: Space Before: 0 pt, After: 0 pt

Data Availability

Raw sequencing data belonging to the 1st EHI data release are available at the European Nucleotide Archive, under Bioproject accession number PRJEB76898, which is nested within the Earth Hologenome Initiative's umbrella Bioproject PRJEB51837. A tarball containing fasta files of all MAGs was deposited in Zenodo [40]under doi: 10.5281/zenodo.16689667. Details of the specific sample and data accession numbers, their associated metadata, as well as the code used for visualisation and summary statistics, can be found in the Github [41]repository https://github.com/earthhologenome/EHI_data_release_1, with a snapshot which was frozen in Zenodo [42]. under doi:10.5281/zenodo.16672754 In addition, the Github repository is also archived in Software Heritage [43]. The overview of all EHI data is available at the EHI database [36]www.earthhologenome.org/database.

Abbreviations

Formatted: Font: Bold

ABS
EHI
ENA
GB
MAG

Declarations

Formatted: Font: Bold

Consent for publication

Not applicable.

Competing interests

The author(s) declare that they have no competing interests.

Funding

Danish National Research Foundation, DNRF (ROR: 00znyv691): DNRF143
Carlsberg Foundation (ROR: 01kpjmx04): CF20-0460
European Commission (ROR: 00k4n6c32) (HORIZON-MSCA-2021-PF-01): 101066225
Fundação para Ciência e Tecnologia (ROR: 00snfqn58): PD/BD/150645/2020
CESAM (ROR: 03q36qm19): LA/P/0094/2020
Villum Fonden (ROR: 05nqkay65): 25925
Fundación Margarita Salas: MS21-053

Authors contributions

NG, RE and AA wrote the manuscript. NG, CP, GMB and JL contributed to the data generation. RE, OA and AA conducted the data analysis. JF and EF collected the *Chalcides striatus* and *Natrix astreptophora* samples. FA, TS and CP collected *Podarcis muralis* samples. GMB collected samples of *Podarcis muralis, Podarcis liolepis* and *Calotriton asper*. LW, CR, MS and CT collected the *Sciurus vulgaris* and *Sciurus carolinensis* samples. AOS, MG and MHW collected the *Perisoreus infaustus* samples. GPL, JA, PA and FC collected *Podarcis muralis* and *Podarcis pityusensis* samples. FC, RG-R and TU collected *Podarcis pityusensis* samples. NF and JA collected *Podarcis filfolensis* samples. NF, JA, GMW and IP collected *Podarcis gaigeae* samples. TU, NF, GMW and IP contributed with *Podarcis milensis* samples. RE collected the *Trichosurus vulpecula* samples. JJ and PA collected the *Plecotus auritus* samples. PH and EB collected *Zoonotrichia capensis* and *Geospizopsis unicolor* samples. PKI and RR collected the *Canis lupus familiaris* samples.

Acknowledgements

The EHI could not be conceived without the trust and economic support provided by the Danish National Research Foundation through the grant DNRF143, and the Carlsberg Foundation through the grant CF20-0460. Additionally, CR was funded by the European Union through an MSCA Postdoctoral Fellowship (HORIZON-MSCA-2021-PF-01; Grant ID: 101066225). Special thanks go to Anders J. Hansen, Head of the Globe Institute, for signing numerous participation agreements, and to project managers Aoife Leonard and Ella Lattenkamp, whose management ensured the smooth handling of the practical aspects of the EHI. The Estación Biológica Cantábrica and Asociación Amigos de Doñana made possible the collection of bat samples. JF was supported by a PhD grant from FCT - Fundação para Ciência e Tecnologia (PD/BD/150645/2020). We also acknowledge financial support to UID Centre for Environmental and Marine Studies (CESAM; LA/P/0094/2020, through national funds. PAH was supported by research grant no. 25925 from VILLUM FONDEN. JA was supported by Margarita Salas contract no. MS21-053 from University of Valencia. PKI and RR were supported by five smaller national funds as well as the Sirius Dog Sled Patrol of Denmark and veterinarian Lone Lykke Hansen.

References

1. Leonard A, Earth Hologenome Initiative Consortium, Alberdi A. A global initiative for ecological and evolutionary hologenomics. *Trends Ecol Evol*. 2024;39 7:616–20.

2. McFall-Ngai M, Hadfield MG, Bosch TCG, Carey HV, Domazet-Lošo T, Douglas AE, et al.. Animals in a bacterial world, a new imperative for the life sciences. *Proc Natl Acad Sci U S A*. 2013;110 9:3229–36.

3. Bordenstein SR, The Holobiont Biology Network, Holobiont Biology Network. The disciplinary matrix of holobiont biology. *Science*. 2024;386 6723:731–2.

4. Alberdi A, Andersen SB, Limborg MT, Dunn RR, Gilbert MTP. Disentangling host–microbiota complexity through hologenomics. *Nat Rev Genet*. 2022;23:281–97.

5. Pietroni C, Gaun N, Leonard A, Lauritsen J, Martin-Bideguren G, Odriozola I, et al.. Hologenomic data generation and analysis in wild vertebrates. *Methods in Ecology and Evolution*. 2025;16 1:97–107.

6. Ellegren H. Genome sequencing and population genomics in non-model organisms. *Trends Ecol Evol*. 2014;29 1:51–63.

7. Taş N, de Jong AE, Li Y, Trubl G, Xue Y, Dove NC. Metagenomic tools in microbial ecology research. *Curr Opin Biotechnol*. 2021;67:184–91.

8. Hernández M, Ancona S, Hereira-Pacheco S, Díaz DE LA Vega-Pérez AH, Navarro-Noya YE. Comparative analysis of two nonlethal methods for the study of the gut bacterial communities in wild lizards. *Integr Zool*. 2023;18 6:1056–71.

9. Ingala MR, Simmons NB, Wultsch C, Krampis K, Speer KA, Perkins SL. Comparing Microbiome Sampling Methods in a Wild Mammal: Fecal and Intestinal Samples Record Different Signals of Host Ecology, Evolution. *Front Microbiol*. 2018;9:803.

10. Kohn MH, York EC, Kamradt DA, Haught G, Sauvajot RM, Wayne RK. Estimating population size by genotyping faeces. *Proc Biol Sci*. 1999;266 1420:657–63.

11. Quince C, Walker AW, Simpson JT, Loman NJ, Segata N. Shotgun metagenomics, from sampling to analysis. *Nat Biotechnol*. 2017;35 9:833–44.

12. Chaumeil P-A, Mussig AJ, Hugenholtz P, Parks DH. GTDB-Tk v2: memory friendly classification with the genome taxonomy database. *Bioinformatics*. 2022;38 23:5315–6.

13. Jain C, Rodriguez-R LM, Phillippy AM, Konstantinidis KT, Aluru S. High throughput ANI analysis of 90K prokaryotic genomes reveals clear species boundaries. *Nat Commun*. 2018;9 1:5114.

14. Pietroni C, Alberdi A: The Earth Hologenome Initiative Laboratory Workflow. In: *The Earth Hologenome Initiative Laboratory Workflow*. 2023. https://www.earthhologenome.org/laboratory Accessed 2023 Jan 12.

15. Carøe C, Gopalakrishnan S, Vinner L, Mak SST, Sinding MHS, Samaniego JA, et al.. Single-tube library preparation for degraded DNA. *Methods Ecol Evol*. 2018;9 2:410–9.

16. Murray DC, Coghlan ML, Bunce M. From benchtop to desktop: important considerations when designing amplicon sequencing workflows. *PLoS One*. 2015;10 4:e0124671.

17. Eisenhofer R, Alberdi A: The Earth Hologenome Initiative Bioinformatics Workflow. In: *The Earth Hologenome Initiative Bioinformatics Workflow*. 2023. https://www.earthhologenome.org/bioinformatics Accessed 2023 Jan 12.

18. UCPH: ERDA: Electronic Data Archive at the University of Copenhagen. 2025. https://erda.dk/ Accessed 2025 Aug 11.

19. Köster J, Rahmann S. Snakemake—a scalable bioinformatics workflow engine. *Bioinformatics*. 2012;28 19:2520–2.

20. Yoo AB, Jette MA, Grondona M. SLURM: Simple Linux Utility for Resource Management. *Job Scheduling Strategies for Parallel Processing*. Berlin, Heidelberg: Springer Berlin Heidelberg; 2003. p. 44–60.

21. Chen S, Zhou Y, Chen Y, Gu J. fastp: an ultra-fast all-in-one FASTQ preprocessor. *Bioinformatics*. 2018;34 17:i884–90.

22. Langmead B, Salzberg SL. Fast gapped-read alignment with Bowtie 2. *Nat Methods*. 2012;9 4:357–9.

23. Li H, Handsaker B, Wysoker A, Fennell T, Ruan J, Homer N, et al.. The Sequence Alignment/Map format and SAMtools. *Bioinformatics*. 2009;25 16:2078–9.

24. Rodriguez-R LM, Gunturu S, Tiedje JM, Cole JR, Konstantinidis KT. Nonpareil 3: Fast Estimation of Metagenomic Coverage and Sequence Diversity. *mSystems*. 2018;3 3. http://dx.doi.org/10.1128/mSystems.00039-18.doi:10.1128/mSystems.00039-18.

25. Woodcroft BJ, Aroney STN, Zhao R, Cunningham M, Mitchell JAM, Blackall L, et al.. SingleM and Sandpiper: Robust microbial taxonomic profiles from metagenomic data. bioRxiv. https://www.biorxiv.org/content/biorxiv/early/2024/01/31/2024.01.30.578060 Accessed 2024 Mar 26.https://doi.org/10.1101/2024.01.30.578060.

26. Eisenhofer R, Alberdi A, Woodcroft BJ. Quantifying microbial DNA in metagenomes improves microbial trait estimation. *ISME Commun*. 2024; https://academic.oup.com/ismecommun/advance-article-abstract/doi/10.1093/ismeco/ycae111/7750770.doi:10.1093/ismeco/ycae111.

27. Li D, Liu C-M, Luo R, Sadakane K, Lam T-W. MEGAHIT: an ultra-fast single-node solution for large and complex metagenomics assembly via succinct de Bruijn graph. *Bioinformatics*. 2015;31 10:1674–6.

28. Alneberg J, Bjarnason BS, de Bruijn I, Schirmer M, Quick J, Ijaz UZ, et al.. Binning metagenomic contigs by coverage and composition. *Nat Methods*. 2014;11 11:1144–6.

29. Wu Y-W, Simmons BA, Singer SW. MaxBin 2.0: an automated binning algorithm to recover genomes from multiple metagenomic datasets. *Bioinformatics*. 2016;32 4:605–7.

30. Kang DD, Li F, Kirton E, Thomas A, Egan R, An H, et al.. MetaBAT 2: an adaptive binning algorithm for robust and efficient genome reconstruction from metagenome assemblies. *PeerJ*. 2019;7:e7359.

31. Gurevich A, Saveliev V, Vyahhi N, Tesler G. QUAST: quality assessment tool for genome assemblies. *Bioinformatics*. 2013;29 8:1072–5.

32. Uritskiy GV, DiRuggiero J, Taylor J. MetaWRAP—a flexible pipeline for genome-resolved metagenomic data analysis. *Microbiome*. 2018;6 1:1–13.

33. Parks DH, Imelfort M, Skennerton CT, Hugenholtz P, Tyson GW. CheckM: assessing the quality of microbial genomes recovered from isolates, single cells, and metagenomes. *Genome Res*. 2015;25 7:1043–55.

34. Parks DH, Chuvochina M, Waite DW, Rinke C, Skarshewski A, Chaumeil P-A, et al.. A standardized bacterial taxonomy based on genome phylogeny substantially revises the tree of life. *Nat Biotechnol*. 2018;36 10:996–1004.

35. Paradis E, Claude J, Strimmer K. APE: Analyses of Phylogenetics and Evolution in R language. *Bioinformatics*. 2004;20 2:289–90.

36. Alberdi A: The Earth Hologenome Initiative Database. In: *The Earth Hologenome Initiative Database*. 2025. www.earthhologenome.org/database Accessed 2025 Aug 11.

37. Aizpurua O, Dunn RR, Hansen LH, Gilbert MTP, Alberdi A. Field and laboratory guidelines for reliable bioinformatic and statistical analysis of bacterial shotgun metagenomic data. *Crit Rev Biotechnol*. 2023;1–19.

38. Bowers RM, Kyrpides NC, Stepanauskas R, Harmon-Smith M, Doud D, Reddy TBK, et al.. Minimum information about a single amplified genome (MISAG) and a metagenome-assembled genome (MIMAG) of bacteria and archaea. *Nat Biotechnol*. 2017;35 8:725–31.

39. Birney E, Hudson T, Green E, Gunter C, Eddy S, Rogers J, et al.. Prepublication data

sharing. *Nature*. 2009;461:168–70.

40. Alberdi, A. (2025). Earth Hologenome Initiative (EHI) Data Release 1: sequence files of metagenome assembled genomes (MAGs) [Data set]. Zenodo. https://doi.org/10.5281/zenodo.16689666Alberdi A. Earth Hologenome Initiative (EHI) Data Release 1: sequence files of metagenome assembled genomes (MAGs). [Data set]. Zenodo. 2025. https://doi.org/10.5281/zenodo.16757580.

41. Alberdi A: Earth Hologenome Initiative Data Release 1. Github. 2025. https://github.com/earthhologenome/EHI_data_release_1.

42. Alberdi, A. (2025). Earth Hologenome Initiative (EHI) Data Release 1: metadata files and analysis code (1.0.3). Zenodo. https://doi.org/10.5281/zenodo.15347437Alberdi A. Earth Hologenome Initiative (EHI) Data Release 1: metadata files and analysis code. Zenodo. 2025. https://doi.org/10.5281/zenodo.16784684.

43. Gaun N, Pietroni C, Martin-Bideguren G, Lauritsen J et al. (2025) The Earth Hologenome Initiative: Data Release 1 (Version 1). [Computer software]. Software Heritage, https://archive.softwareheritage.org/swh:1:snp:c4861440bd494ef8ab8c9d4390d1492a934501f6 Alberdi A. The Earth Hologenome Initiative: Data Release 1 (Version 1). [Computer software]. Software Heritage. https://archive.softwareheritage.org/browse/snapshot/2b5e0842ffeeb3f66eb669c74ee78289202b6080/directory/ Accessed 2025 Aug 11.

Formatted: Font color: Auto

Formatted: Font color: Auto

Formatted: Font color: Auto

Tables

Table 1. Summary statistics of the animal species represented in the 1st EHI data release. Detailed metadata tables are available as part of the supporting files.

Species	Taxonomy	Sampling events	Individuals	Samples	Data (GB)	Genomes	Percentage new
Calotriton asper	Urodela, Amphibia	5	31	37	230.4	745	95.0
Canis lupus familiaris	Carnivora, Mammalia	14	58	58	333.7	1252	39.3
Chalcides striatus	Squamata, Reptilia	2	2	2	39.5	0	-
Geospizopsis unicolor	Passeriformes, Aves	1	2	2	18.3	0	-
Lepus europaeus	Lagomorpha, Mammalia	15	25	50	252.6	711	85.4
Lissotriton helveticus	Urodela, Amphibia	16	88	97	444.7	1590	95.9
Natrix astreptophora	Squamata, Reptilia	2	2	2	32.8	0	-
Perisoreus infaustus	Passeriformes, Aves	2	2	2	32.5	0	-
Plecotus auritus	Chiroptera, Mammalia	1	2	2	42.1	0	-
Podarcis filfolensis	Squamata, Reptilia	9	43	43	174.7	693	91.9
Podarcis gaigeae	Squamata, Reptilia	17	61	61	303.5	1280	97.3
Podarcis liolepis	Squamata, Reptilia	2	13	13	67.0	232	92.2
Podarcis milensis	Squamata, Reptilia	8	26	26	149.7	590	96.6
Podarcis muralis	Squamata, Reptilia	35	154	165	998.5	2670	97.5
Podarcis pityusensis	Squamata, Reptilia	12	43	43	220.8	1046	93.1
Psittacula echo	Psittaciformes, Aves	49	48	50	591.2	123	53.6
Salamandra atra	Urodela, Amphibia	1	2	2	23.8	0	-
Sciurus carolinensis	Rodentia, Mammalia	47	65	120	533.1	1686	95.8

Sciurus vulgaris	Rodentia, Mammalia	76	74	123	660.5	1033	72.3
Trichosurus vulpecula	Diprotodontia, Mammalia	2	2	2	20.9	61	88.5
Zonotrichia capensis	Passeriformes, Aves	1	2	2	28.0	0	-

Figures

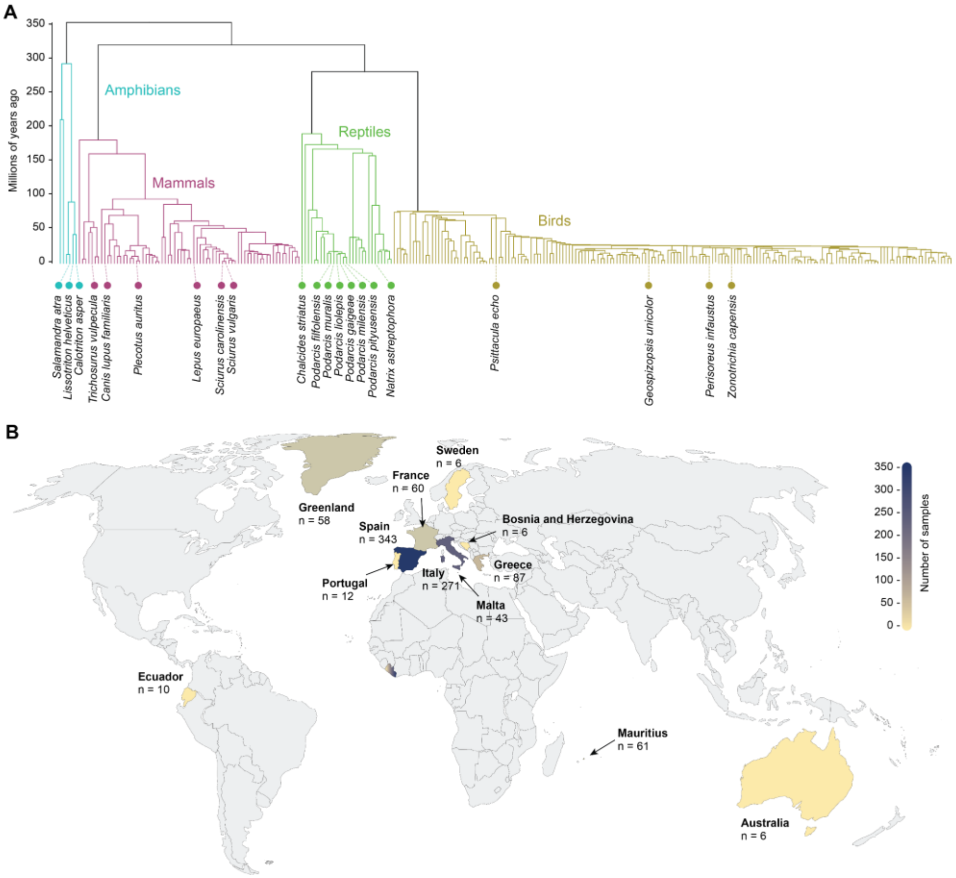

Figure 1. **Phylogenetic placement and geographic origin of the samples**. **A**) Phylogenetic tree of all vertebrate species represented in the EHI collection in 2025 Q1, with the phylogenetic position of the species included in this data release highlighted. **B**) World map indicating the number of samples sourced from each of the represented countries.

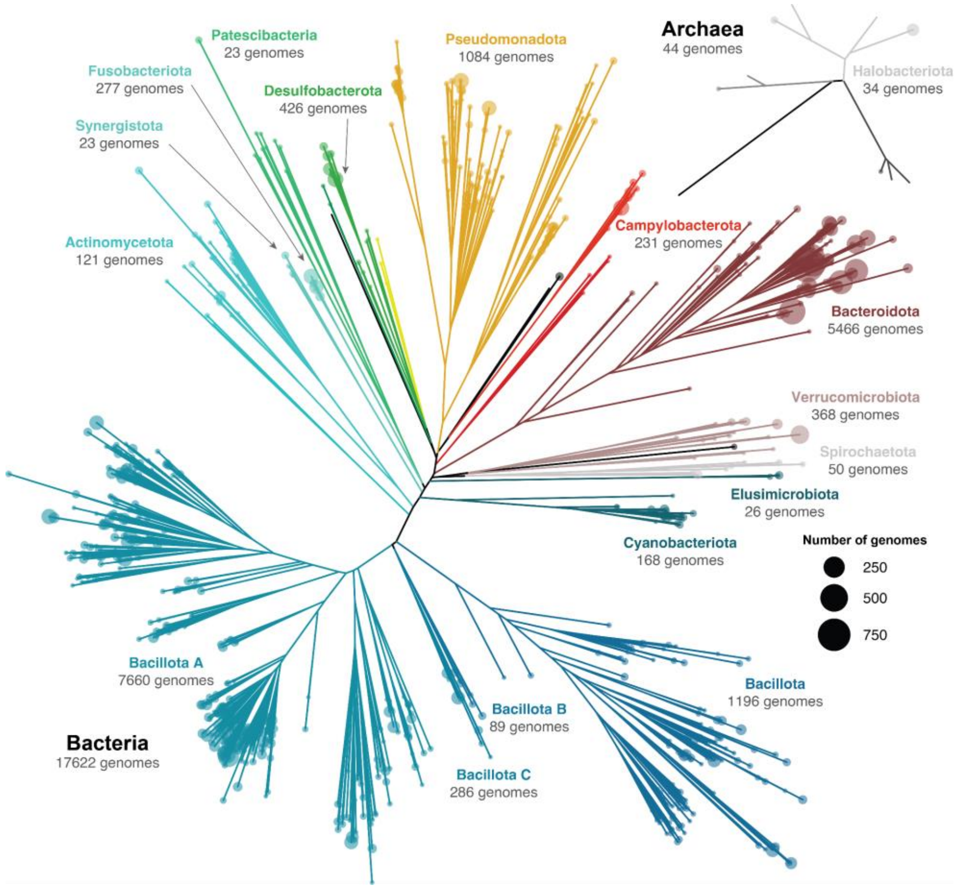

Figure 2. **Phylogenetic trees of the EHI-reconstructed bacterial and archaeal genomes.** Each tip represents a genus and the tip size indicates the number of released genomes within the genus.

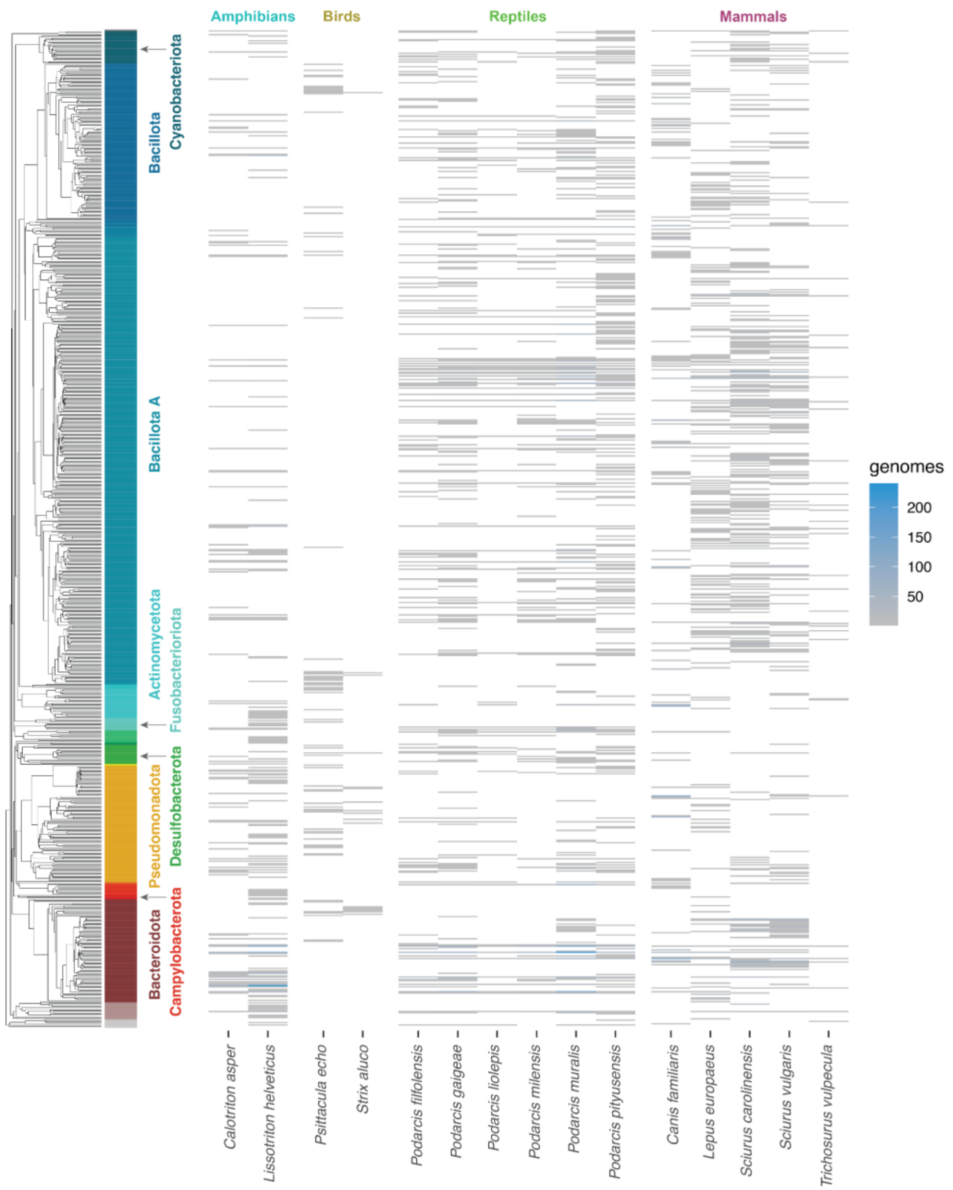

Figure 3. **Host breadth of the reconstructed bacterial taxa.** Only genomes reconstructed from individual assemblies are displayed in this figure. *Chalcides striatus*, *Geospizopsis unicolor*, *Natrix astreptophora*, *Plecotus auritus*, *Salamandra atra*, and *Zonotrichia capensis* did not yield any metagenome-assembled genomes from individual assemblies. Note that only the

most abundant bacterial phylum names are displayed for the sake of visualisation. Exact data can be found in the supplementary materials.

Figure 1

Click here to access/download;Figure;figure1.png

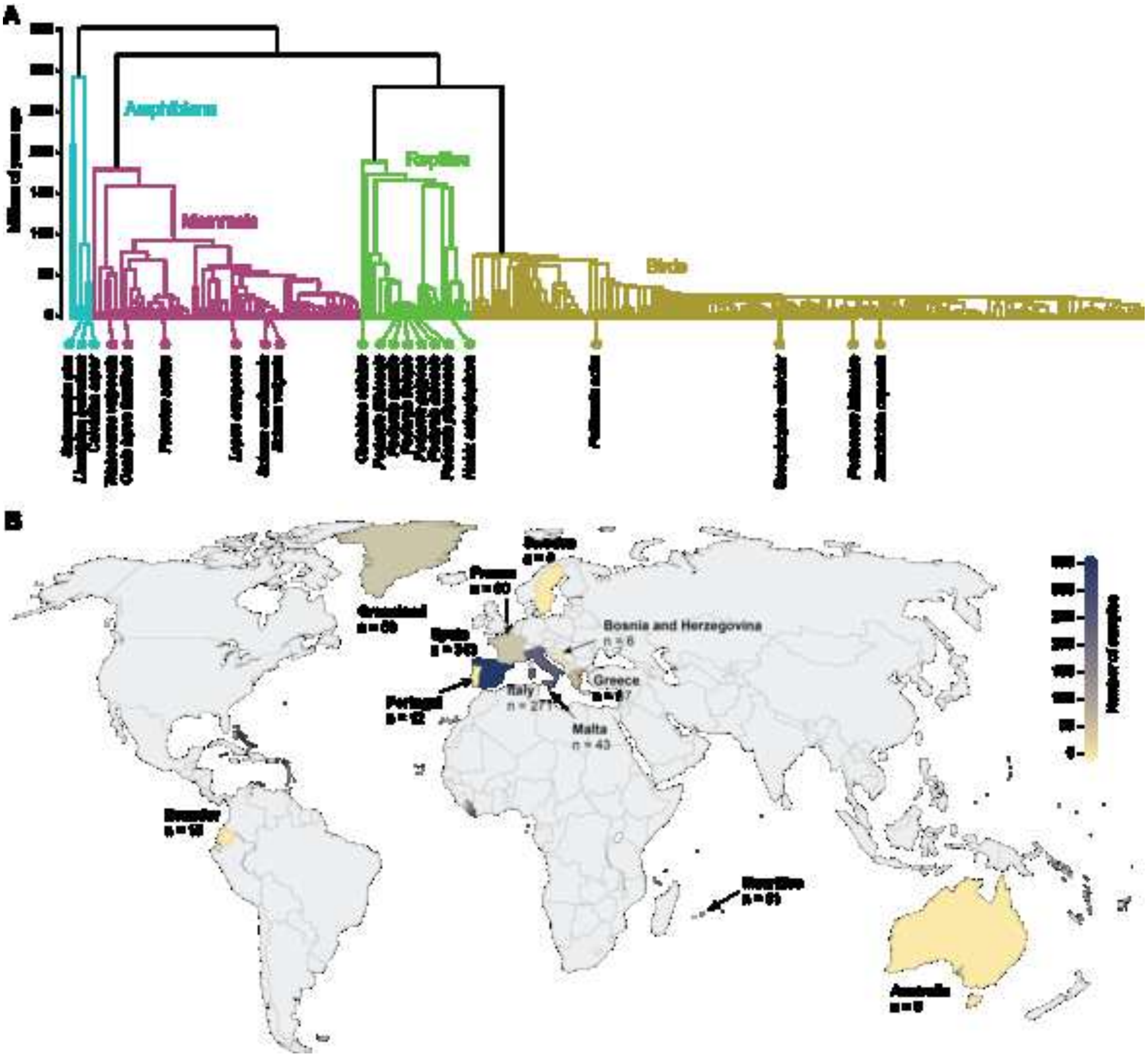

Figure 2

Click here to access/download;Figure;figure2.png

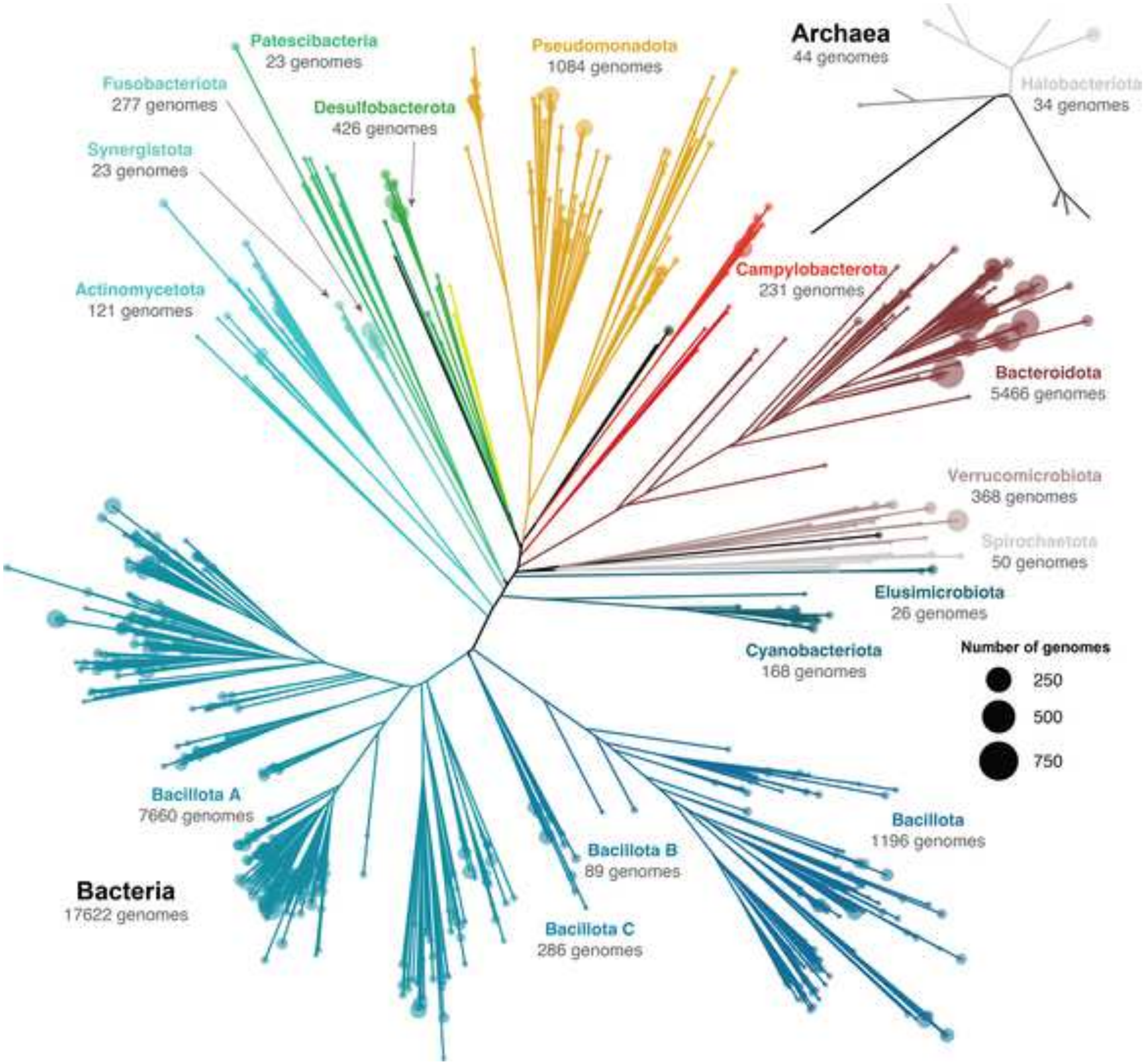

Figure 3

Click here to access/download;Figure;figure3.png

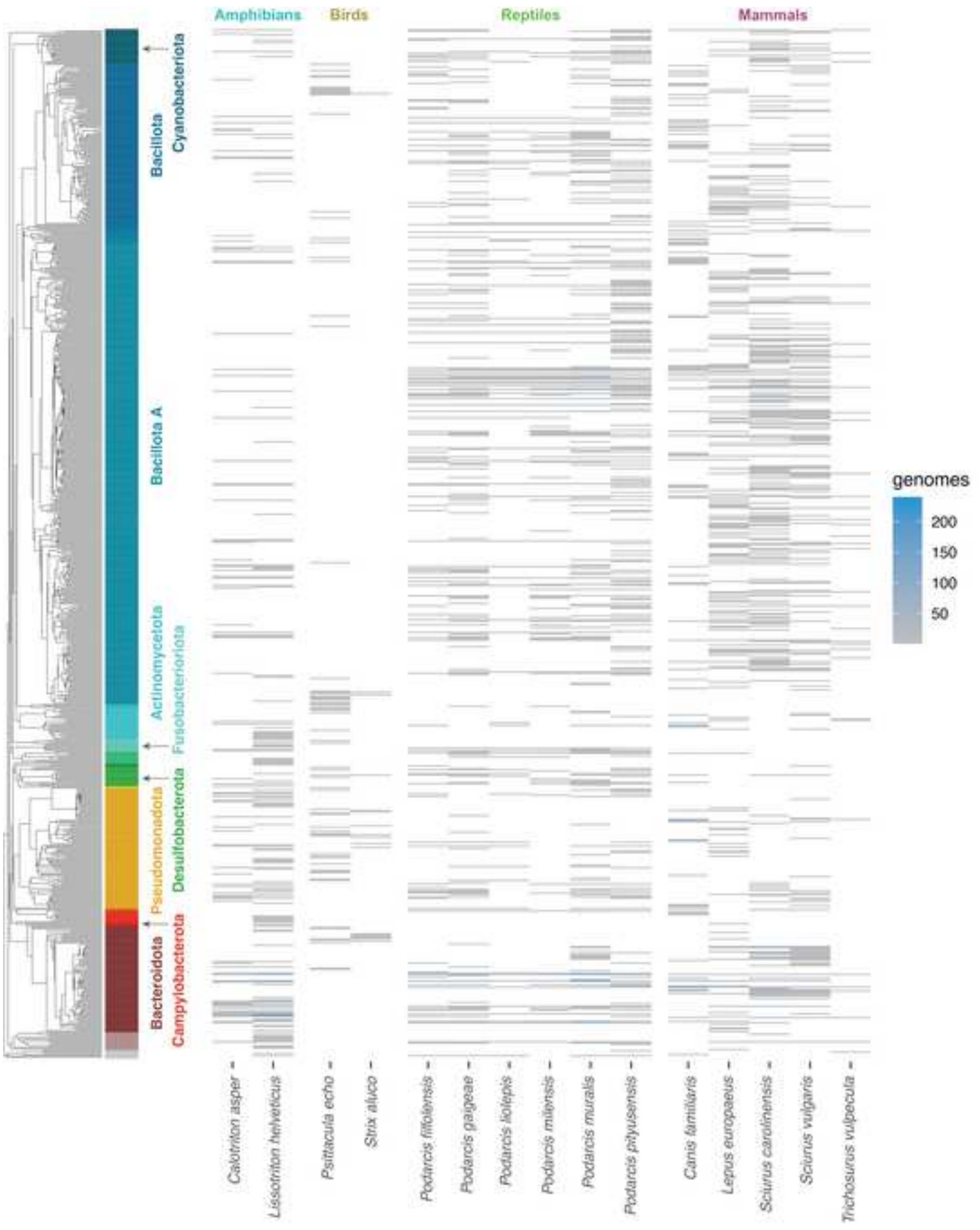

Supplement: giaf102_GIGA-D-25-00196_Revision_2 [file giaf102_giga-d-25-00196_revision_2.pdf]
