# Supplementary material for: The Earth Hologenome Initiative: Data Release 1
Source: Gigascience. 2025 Sep 5;14:giaf102. doi: 10.1093/gigascience/giaf102 (PMC12412122; doi:10.1093/gigascience/giaf102)
Supplement: giaf102_Authors_Response_To_Reviewer_Comments_Original_Submission [file giaf102_authors_response_to_reviewer_comments_original_submission.pdf]

Author's Response To Reviewer Comments

First of all, we would like to thank the editor and the reviewers for their positive feedback on our manuscript. We have incorporated the requested changes in the main manuscripts, and we have also modified the bioinformatic code to account for the extra analysis requested by the reviewers. We hope that the updated manuscript and associated resources meet the requirements of GigaScience and our manuscript gets accepted for publication.

Reviewer #1: The manuscript is well written and represents a fantastic dataset. I do not have many comments.

>> We very much appreciate the positive feedback.

Are the methods appropriate to the aims of the study, are they well described, and are necessary controls included? Yes. The methodology for sample collection is clear. Controls were described adequately. Although not required for this dataset, I recommend that future sequencing efforts include a positive control such as ZymoBIOMICS Microbial Community Standards, as this will greatly improve the comparisons across different sequencing batches.

>> We have included further information on the use of controls in the laboratory processing of the samples.

Line 112-113: "The released data include 6.4% of low-quality DNA, 19.1% of DNA mapped to host genomes, and 74.5% of metagenomic DNA." is this as a whole or average per sample? Could you please also include the average DNA mapped per host genome and metagenomic DNA including standard error?

>> We have now calculated the per-sample relative data statistics and reported the mean and standard deviation values in the manuscript. The code for conducting such calculations has been also updated in the repository.

Line 159: Could you please specify the number of sequencing batches? Is the sequencing batch reported in the metadata? If not, please include.

>> We have now included the number of batches, and we have included the batch code in the “raw data” table of the metadata.

Line 216: missing space between "DNAto"

>> Fixed.

Reviewer #2: The Earth Hologenome Initiative: Data Release 1 by Gaun et al. presents an important advancement in the availability of hologenomic resources from several wild animal species. The authors provide a detailed description of the standardized procedures used across all stages of the workflow, including field sampling, wet-lab protocols (e.g., DNA extraction and sequencing library preparation), and dry-lab processes (e.g., bioinformatic analyses from quality control to metagenome-assembled genome reconstruction). These resources represent a significant contribution to the study of ecological and evolutionary processes in animals. Moreover, they offer a valuable foundation for developing conservation strategies aimed at protecting biodiversity and natural habitats.

>> Thank you very much for the positive feedback.

1. The manuscript does not mention whether routine quality control procedures, such as the use of positive and negative controls, were implemented in the laboratory workflows. It would be helpful to clarify if blank controls (e.g., from reagents) or mock communities were included to validate the DNA extraction and sequencing protocols.

>> We have now added further information on the use of controls and batch construction strategies.

2. The manuscript provides a comprehensive description of the bioinformatics workflows applied to microbial analysis, including access to all relevant scripts in the provided links. However, since a hologenome also encompasses the host's genomic data, it would be beneficial to include details regarding the bioinformatic processing of host genomes. Specifically, workflows for genome assembly, phylogenetic analysis, and SNP calling (if applicable) are not described or made available, even though host data are mentioned as included.

>> While we have developed our own bioinformatic pipelines for host genotyping (e.g., https://github.com/alberdilab/genotyping), these are not routinely applied to EHI data the same way the genome-resolved metagenomic pipeline is applied, as the optimal analysis depends largely on data structure. The routine pipeline is limited to mapping the reads to the host genome, which is already described in the manuscript.

L135. The reference is missing

>> We have included the reference.

Editorial Manager®

Cookie Preference Center

We use cookies which are necessary to make our site work. We may also use additional cookies to analyze, improve and personalize our content and your digital experience. For more information, see our Cookie Policy.

You may choose not to allow some types of cookies. However, blocking some types may impact your experience of our site and the services we are able to offer. See the different category headings below to find out more or change your settings.

Allow all

Manage Consent Preferences

Strictly Necessary Cookies

Always active

These cookies are necessary for the website to function and cannot be switched off in our systems. They are usually only set in response to actions made by you which

amount to a request for services, such as setting your privacy preferences, logging in or filling in forms. You can set your browser to block or alert you about these cookies, but some parts of the site will not then work.

Functional Cookies

These cookies enable the website to provide enhanced functionality and personalisation. They may be set by us or by third party providers whose services we have added to our pages. If you do not allow these cookies then some or all of these services may not function properly.

Performance Cookies

These cookies allow us to count visits and traffic sources so we can measure and improve the performance of our site. They help us to know which pages are the most and least popular and see how visitors move around the site.

Targeting Cookies

These cookies may be set through our site by our advertising partners. They may be used by those companies to build a profile of your interests and show you relevant adverts on other sites. If you do not allow these cookies, you will experience less targeted advertising.

Cookie List

Clear

Apply Cancel

Consent Leg.Interest

Confirm my choices

Powered by onetrust
