# Supplementary material for: The Earth Hologenome Initiative: Data Release 1
Source: Gigascience. 2025 Sep 5;14:giaf102. doi: 10.1093/gigascience/giaf102 (PMC12412122; doi:10.1093/gigascience/giaf102)
Supplement: giaf102_Reviewer_1_Report_Original_Submission — Chuen Zhang Lee -- 6/6/2025 [file giaf102_reviewer_1_report_original_submission.pdf]

Reviewer Report

Title: The Earth Hologenome Initiative: Data Release 1

Version: Original Submission **Date:** 6/6/2025

Reviewer name: Chuen Zhang Lee

Reviewer Comments to Author:

The manuscript is well written and represents a fantastic dataset. I do not have many comments.1) Are the methods appropriate to the aims of the study, are they well described, and are necessary controls included?Yes. The methodology for sample collection is clear. Controls were described adequately. Although not required for this dataset, I recommend that future sequencing efforts include a positive control such as ZymoBIOMICS Microbial Community Standards, as this will greatly improve the comparisons across different sequencing batches.2) Are the conclusions adequately supported by the data shown?Yes. Good conclusions.3) Please indicate the quality of language in the manuscript. Does it require a heavy editing for language and clarity?Good quality of language.4) Are you able to assess all statistics in the manuscript, including the appropriateness of statistical tests used?There were no statistical tests used in this manuscript.Reviewer comments:1. Line 112-113: "The released data include 6.4% of low-quality DNA, 19.1% of DNA mapped to host genomes, and 74.5% of metagenomic DNA." is this as a whole or average per sample? Could you please also include the average DNA mapped per host genome and metagenomic DNA including standard error?2. Line 159: Could you please specify the number of sequencing batches?Is the sequencing batch reported in the metadata? If not, please include.3. Line 216: missing space between "DNAto"

Level of Interest

Please indicate how interesting you found the manuscript: Choose an item.

Quality of Written English

Please indicate the quality of language in the manuscript: Choose an item.

Declaration of Competing Interests

Please complete a declaration of competing interests, considering the following questions:

- Have you in the past five years received reimbursements, fees, funding, or salary from an organisation that may in any way gain or lose financially from the publication of this manuscript, either now or in the future?
- Do you hold any stocks or shares in an organisation that may in any way gain or lose financially from the publication of this manuscript, either now or in the future?

- Do you hold or are you currently applying for any patents relating to the content of the manuscript?
- Have you received reimbursements, fees, funding, or salary from an organization that holds or has applied for patents relating to the content of the manuscript?
- Do you have any other financial competing interests?
- Do you have any non-financial competing interests in relation to this paper?

If you can answer no to all of the above, write 'I declare that I have no competing interests' below. If your reply is yes to any, please give details below.

I declare that I have no competing interests

I agree to the open peer review policy of the journal. I understand that my name will be included on my report to the authors and, if the manuscript is accepted for publication, my named report including any attachments I upload will be posted on the website along with the authors' responses. I agree for my report to be made available under an Open Access Creative Commons CC-BY license (http://creativecommons.org/licenses/by/4.0/). I understand that any comments which I do not wish to be included in my named report can be included as confidential comments to the editors, which will not be published.

Choose an item.

To further support our reviewers, we have joined with Publons, where you can gain additional credit to further highlight your hard work (see: https://publons.com/journal/530/gigascience). On publication of this paper, your review will be automatically added to Publons, you can then choose whether or not to claim your Publons credit. I understand this statement.

Yes Choose an item.
