# Supplementary material for: The Earth Hologenome Initiative: Data Release 1
Source: Gigascience. 2025 Sep 5;14:giaf102. doi: 10.1093/gigascience/giaf102 (PMC12412122; doi:10.1093/gigascience/giaf102)
Supplement: giaf102_Reviewer_2_Report_Original_Submission — Yendi E Navarro Noya -- 7/30/2025 [file giaf102_reviewer_2_report_original_submission.pdf]

Reviewer Report

Title: The Earth Hologenome Initiative: Data Release 1

Version: Original Submission **Date:** 7/30/2025

Reviewer name: Yendi E Navarro Noya

Reviewer Comments to Author:

The Earth Hologenome Initiative: Data Release 1 by Gaun et al. presents an important advancement in the availability of hologenomic resources from several wild animal species. The authors provide a detailed description of the standardized procedures used across all stages of the workflow, including field sampling, wet-lab protocols (e.g., DNA extraction and sequencing library preparation), and dry-lab processes (e.g., bioinformatic analyses from quality control to metagenome-assembled genome reconstruction). These resources represent a significant contribution to the study of ecological and evolutionary processes in animals. Moreover, they offer a valuable foundation for developing conservation strategies aimed at protecting biodiversity and natural habitats.Minor comments:1. The manuscript does not mention whether routine quality control procedures, such as the use of positive and negative controls, were implemented in the laboratory workflows. It would be helpful to clarify if blank controls (e.g., from reagents) or mock communities were included to validate the DNA extraction and sequencing protocols.2. The manuscript provides a comprehensive description of the bioinformatics workflows applied to microbial analysis, including access to all relevant scripts in the provided links. However, since a hologenome also encompasses the host's genomic data, it would be beneficial to include details regarding the bioinformatic processing of host genomes. Specifically, workflows for genome assembly, phylogenetic analysis, and SNP calling (if applicable) are not described or made available, even though host data are mentioned as included.L135. The reference is missing

Level of Interest

Please indicate how interesting you found the manuscript: Choose an item.

Quality of Written English

Please indicate the quality of language in the manuscript: Choose an item.

Declaration of Competing Interests

Please complete a declaration of competing interests, considering the following questions:

- Have you in the past five years received reimbursements, fees, funding, or salary from an organisation that may in any way gain or lose financially from the publication of this manuscript, either now or in the future?

- Do you hold any stocks or shares in an organisation that may in any way gain or lose financially from the publication of this manuscript, either now or in the future?
- Do you hold or are you currently applying for any patents relating to the content of the manuscript?
- Have you received reimbursements, fees, funding, or salary from an organization that holds or has applied for patents relating to the content of the manuscript?
- Do you have any other financial competing interests?
- Do you have any non-financial competing interests in relation to this paper?

If you can answer no to all of the above, write 'I declare that I have no competing interests' below. If your reply is yes to any, please give details below.

I declare that I have no competing interests

I agree to the open peer review policy of the journal. I understand that my name will be included on my report to the authors and, if the manuscript is accepted for publication, my named report including any attachments I upload will be posted on the website along with the authors' responses. I agree for my report to be made available under an Open Access Creative Commons CC-BY license (http://creativecommons.org/licenses/by/4.0/). I understand that any comments which I do not wish to be included in my named report can be included as confidential comments to the editors, which will not be published.

Choose an item.

To further support our reviewers, we have joined with Publons, where you can gain additional credit to further highlight your hard work (see: https://publons.com/journal/530/gigascience). On publication of this paper, your review will be automatically added to Publons, you can then choose whether or not to claim your Publons credit. I understand this statement.

Yes Choose an item.
